# Autophagic receptor p62 protects against glycation‐derived toxicity and enhances viability

**DOI:** 10.1111/acel.13257

**Published:** 2020-11-04

**Authors:** Gemma Aragonès, Kalavathi Dasuri, Opeoluwa Olukorede, Sarah G. Francisco, Carol Renneburg, Caroline Kumsta, Malene Hansen, Shun Kageyama, Masaaki Komatsu, Sheldon Rowan, Jonathan Volkin, Michael Workman, Wenxin Yang, Paula Daza, Diego Ruano, Helena Dominguez‐Martín, José Antonio Rodríguez‐Navarro, Xue‐Liang Du, Michael A. Brownlee, Eloy Bejarano, Allen Taylor

**Affiliations:** ^1^ Laboratory for Nutrition and Vision Research USDA Human Nutrition Research Center on Aging Tufts University Boston MA USA; ^2^ Sanford Burnham Prebys Medical Discovery Institute La Jolla CA USA; ^3^ Department of Physiology Juntendo University School of Medicine Bunkyo Japan; ^4^ Departamento Biología Celular. Facultad de Biología Universidad de Sevilla Sevilla Spain; ^5^ Departamento de Bioquímica y Biología Molecular. Facultad de Farmacia Universidad de Sevilla Sevilla Spain; ^6^ Instituto de Biomedicina de Sevilla (IBiS Hospital Universitario Virgen del Rocío/Consejo Superior de Investigaciones Científicas/Universidad de Sevilla Sevilla Spain; ^7^ Servicio de Neurobiología Departamento de Investigación Hospital Ramón y Cajal Instituto Ramón y Cajal de Investigaciones Sanitarias Carretera de Colmenar Madrid Spain; ^8^ Albert Einstein College of Medicine Bronx NY USA; ^9^ School of Health Sciences Universidad CEU Cardenal Herrera Valencia Spain

**Keywords:** aging, autophagy, glycative stress, p62, proteotoxicity

## Abstract

Diabetes and metabolic syndrome are associated with the typical American high glycemia diet and result in accumulation of high levels of advanced glycation end products (AGEs), particularly upon aging. AGEs form when sugars or their metabolites react with proteins. Associated with a myriad of age‐related diseases, AGEs accumulate in many tissues and are cytotoxic. To date, efforts to limit glycation pharmacologically have failed in human trials. Thus, it is crucial to identify systems that remove AGEs, but such research is scanty. Here, we determined if and how AGEs might be cleared by autophagy. Our *in vivo* mouse and *C*. *elegans* models, in which we altered proteolysis or glycative burden, as well as experiments in five types of cells, revealed more than six criteria indicating that p62‐dependent autophagy is a conserved pathway that plays a critical role in the removal of AGEs. Activation of autophagic removal of AGEs requires p62, and blocking this pathway results in accumulation of AGEs and compromised viability. Deficiency of p62 accelerates accumulation of AGEs in soluble and insoluble fractions. p62 itself is subject to glycative inactivation and accumulates as high mass species. Accumulation of p62 in retinal pigment epithelium is reversed by switching to a lower glycemia diet. Since diminution of glycative damage is associated with reduced risk for age‐related diseases, including age‐related macular degeneration, cardiovascular disease, diabetes, Alzheimer's, and Parkinson's, discovery of methods to limit AGEs or enhance p62‐dependent autophagy offers novel potential therapeutic targets to treat AGEs‐related pathologies.

## Graphical abstract

AGEs are toxic compounds formed by non‐enzymatic reactions between sugars and proteins. AGEs are prone to aggregate. Insoluble AGEs are efficiently removed via p62‐selective autophagy. The autophagic removal of AGEs is a conserved pathway, and the lack of p62 leads to accumulation of toxic AGEs in mouse and worms. Enhanced autophagy is protective against glycation‐derived damage. p62‐dependent autophagy offers novel potential therapeutic targets to treat AGEs‐related pathologies.

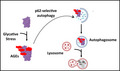

## 1 INTRODUCTION

Americans consume very high glycemic diets, and the trend toward consuming these diets is increasing throughout the world. Associated with consumption of such high glycemic diets are markedly increased risks for many major age‐related debilities including cardiovascular disease (CVD), diabetes, age‐related macular degeneration (AMD), some forms of cataract, and neurodegenerative diseases such as Alzheimer's disease and Parkinson's disease (Bejarano & Taylor, 2019; Chaudhuri et al., 2018; Moldogazieva & Mokhosoev, 2019; Vicente Miranda et al., 2016). Alarmingly, the increase in risk for disease due to consuming high glycemic diets is comparable to the risk incurred by smoking. In contrast, consuming lower glycemic diets is associated with slower progression of some of these diseases. These data suggest that switching to lower glycemic diets can reduce the risk of developing several severe medical conditions, bringing tremendous personal and public health benefits. What might be mechanisms for the salutary effect of lower glycemic diets?

Protein glycation results from the non‐enzymatic chemical reaction of sugars with proteins. Initial steps are called the Amadori and Maillard reactions. Metabolic products of sugar, some oxidized, such as methylglyoxal (MGO) are primary biological glycating agents. The products can progress through a myriad of rearrangements and additional reactions. Collectively, these are called advanced glycation end products (AGEs). The excess glucose and its metabolic products that result from a high glycemia diet, or diabetes, have been shown to induce and accelerate glycative stress. Even in nondiabetics, AGEs accumulate with accelerating rates upon aging in most tissues, in pathologies such as cataracts and AMD and are cytotoxic (Kazi et al., 2017; Rabbani & Thornalley, 2015; Uchiki et al., 2012; Weikel et al., 2012). We and others have observed elevated levels of AGEs in tissues, including liver, brain, retina, heart, collagen, of nondiabetic animals that consumed high glycemic diets or were aged (Uchiki et al., 2012; Weikel et al., 2012). In contrast, diminishing the level of AGEs has been proven to prolong lifespan in model organisms (Kazi et al., 2017). Efficient removal of AGEs is especially relevant in highly differentiated tissues such as the retina, lens or brain. In such organs, glycation damage cannot be diluted by cellular division and is indicative of disease (Chaudhuri et al., 2018).

The mechanisms by which AGEs damage the individual are poorly understood. It has been proposed that AGEs threaten cellular homeostasis by compromising the function of critical biomolecules, forming dysfunctional toxic aggregates, and recruiting and/or inactivating other essential proteins. Collectively, these insults lead to aberrant metabolism and cellular vulnerability (Rabbani & Thornalley, 2015).

The deposition of AGEs can be limited by detoxification of reactive AGEs precursors such as MGO via the glyoxalase system (Morcos et al., 2008). However, once AGEs are formed, there are no enzymes that specifically remove the added sugar or sugar derivatives from proteins. Two major proteolytic pathways are proposed to contribute to the AGEs clearance: the ubiquitin‐proteasome system (UPS) and autophagy (Takahashi et al., 2017; Taylor, 2012). The UPS operates mainly on soluble substrates and uses the proteasome for degradation, whereas autophagy targets cargoes to the lysosomal compartment for degradation. Autophagy is the major degradative route for the clearance of cytosolic, aggregated, or insoluble proteins and organelles that cannot pass through the proteasome.

The UPS and autophagy cooperate functionally, and the deficiency of one of these pathways can trigger the upregulation of the other route (Gavilan et al., 2015; Ji & Kwon, 2017). The function of these degradative pathways declines with age, contributing to the intracellular accumulation of proteinaceous aggregates and dysfunctional organelles in aged tissues (Mizushima et al., 2008). It is presently unknown 1) if the clearance of AGEs is impacted by crosstalk between the UPS and autophagy, 2) if different pools of AGEs are differentially targeted to each pathway, and 3) if upregulating proteolytic pathways increases clearance of AGEs to benefit cell and organismic viability in the face of glycative stress.

During the autophagic process, damaged proteins/organelles or aggregates are tagged with ubiquitin and sequestered into double‐membrane structures called autophagosomes. The completion of this process requires the collaboration of a set of autophagic proteins including structural elements involved in the biogenesis of the autophagosome. These include the microtubule‐associated protein chain 3 (LC3, a mammalian homologue of yeast Atg8) and receptors that target ubiquitinated cargo to the autophagic compartment. The best studied of these autophagic receptors is p62/SQSTM1/A170/ZIP (hereafter called p62). p62 facilitates selective autophagic clearance of protein aggregates and has a crucial function in cellular homeostasis (Johansen & Lamark, 2011; Komatsu et al., 2007; Matsumoto et al., 2011; Pankiv et al., 2007). p62 has binding sites for both ubiquitin and LC3, thus serving as a scaffold (Johansen & Lamark, 2011). Mature autophagosomes fuse with lysosomes that provide digestive enzymes, and both LC3 and p62 are degraded by lysosomal proteases along with the cargo.

Given the apparent deleterious impact of AGEs, there is a need to develop strategies to counteract their accumulation and the disease‐related sequelae. There is limited mechanistic information about the targeting of AGEs for degradation, and a lack of understanding about the role of autophagy in this process limits our ability to formulate therapeutic strategies to reduce the risk for AGEs‐related diseases. In this study, we explored the contribution of p62‐dependent autophagy to the clearance of AGEs. We show for the first time a protective role for p62 against glycation‐derived toxicity and identify this autophagic receptor as a novel potential therapeutic target to treat AGEs‐related pathologies.

## 2 RESULTS

### 2.1 p62‐dependent autophagy plays a role in the clearance of endogenous AGEs

**Figure 1:**
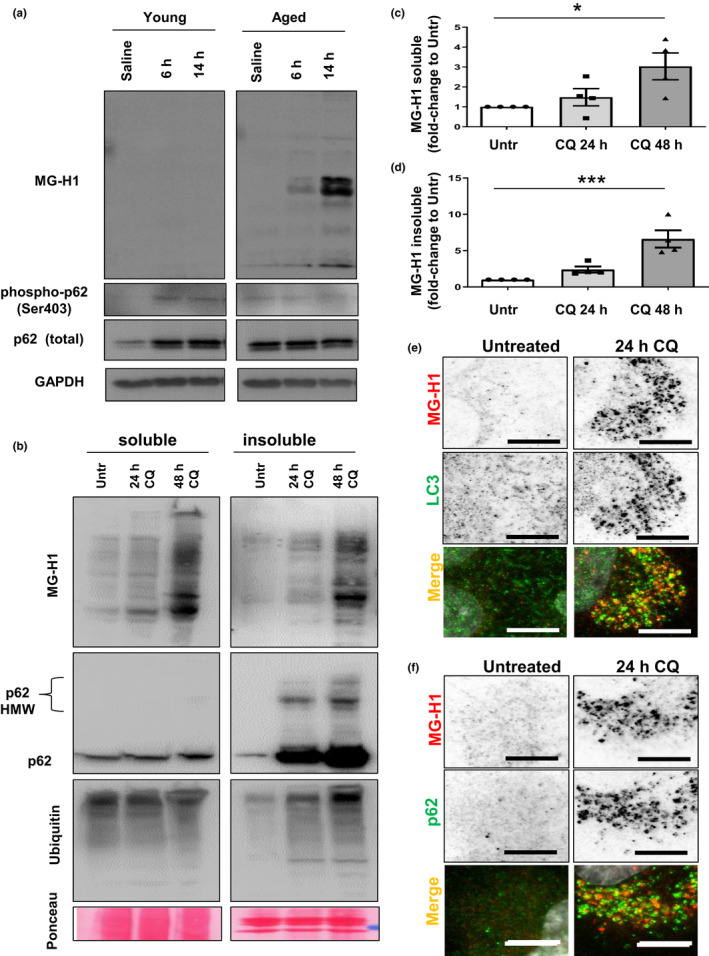
Suppression of lysosomal degradation leads to accumulation of endogenous AGEs in autophagosomes. (a) Representative Western blots for MG‐H1 in young (3‐4 months old) and aged (24‐26 months old) rat hippocampus after proteasome inhibition. Note the increased amount of MG‐H1 in the aged group at 6 h and 14 h after lactacystin‐injection. p62 and phospho‐p62 are shown as autophagic markers and GAPDH as loading control. (b) ARPE‐19 maintained in the presence or absence of CQ for either 24 or 48 h were subjected to extraction with 1% Triton X‐100. Soluble (*left*) and insoluble (*right*) fractions were immunoblotted for the indicated proteins. (C,D) Quantification of total soluble (c) and insoluble (d) MG‐H1 relative to values in untreated cells. Values are mean ±SEM (n = 4). *p < 0.05 and ***p < 0.001 in one‐way ANOVA followed by Dunnett's multiple comparison test. (e,f) Accumulation of MG‐H1 in autophagosomes. HLECs were maintained in the presence or absence of CQ for 24 h, fixed in cold methanol. (e) anti‐LC3 (green) or (f) anti‐p62 (green) was used to stain autophagosomes along with anti MG‐H1 (red) to detect endogenous AGEs. Red and green channels are shown in black and white in the upper panels for a better visualization. Full fields for panel e and f are shown in *SI Appendix* Figure S1c (for LC3) and S2A (for p62). Scale bar: 10 μm

Given that the UPS and autophagy are functionally coupled and are proposed to participate in the removal of AGEs (Taylor, 2012), we first evaluated the importance of these pathways in AGEs clearance *in vivo* by monitoring levels of MGO‐derived hydroimidazolone 1 (MG‐H1), one of the most abundant AGEs. Young (3‐4 months old) and old (24‐26 months old) rats were injected in the hippocampus with the UPS inhibitor lactacystin (Gavilan et al., 2015), and the expression of MG‐H1 was analyzed. In the absence of UPS inhibitor, we observed no significant levels of MG‐H1‐AGEs, hereafter called AGEs, in the hippocampus from young rats and limited AGEs in old rats (Figure 1a, lanes 1,4). However, there was a significant accumulation of AGEs in old rats when the UPS was inhibited *in vivo* (Figure 1a, lanes 5,6 versus 2,3). These data indicate that 1) the combined UPS and autophagic capacity are largely operational at both ages, albeit, 2) AGEs‐degrading UPS activity is limited in the older rats. 3) That there is no accumulation of AGEs in UPS‐inhibited tissue in young animals is consistent with autophagy compensating for the pharmacological reduction of UPS activity in young but not in old rats (Figure 1a, lanes 5, 6 versus lanes 2, 3) (Gavilan et al., 2015).

We previously found that lysosomal activity was involved in clearance of AGEs, some of which were ubiquitinated, but the mechanism of this process remained an enigma (Uchiki et al., 2012). p62 is an autophagic receptor that recruits ubiquitinated substrates to autophagosomes for subsequent degradation in the autolysosome (Pankiv et al., 2007). This suggested the hypothesis that p62 plays a role in AGEs clearance. A role for p62‐dependent autophagy in removal of AGEs is further suggested by the observation that the autophagic receptor p62 and its active phosphorylated form Ser403 (Matsumoto et al., 2011) are upregulated in hippocampus *in vivo* at 6 h in young, but not in aged, rats (Figure 1a, lane 2, 3 versus lane 1). The observed higher levels of total p62 (Figure 1a, lane 4 versus lane 1), yet a reduced extent of phosphorylation (Figure 1a, lanes 5, 6 versus 4), indicate that autophagy is not upregulated in older animals (Gavilan et al., 2015).

A vast literature indicates accumulation mainly in the insoluble fraction of autophagic cargoes when p62‐selective autophagy is impaired (Komatsu et al., 2007), but this has not been explored with regard to AGEs. The retina and lens accumulate AGEs with age as their proteolytic capacities decline (Uchiki et al., 2012). We observed increased levels of AGEs in cells derived from these organs upon prolonged lysosomal blockage by exposure to chloroquine (CQ), which inhibits autophagosome‐lysosome fusion and lysosomal acidification without negatively affecting UPS function (Figure 1b, Figure S1a,b) (Wang et al., 2013). High levels of endogenous AGEs were observed in the soluble and insoluble fractions in epithelial cells from retina and lens when autophagy was blocked with CQ and reached significance earliest in the insoluble fraction (Figure 1b‐d and Figure S1a,b). As expected, p62 and ubiquitin conjugates accumulated in both cell types in the presence of CQ, with larger increases in the insoluble fraction (Figure 1b, Figure S1a,b).

Although found in insoluble fractions, AGEs have not been previously found in autophagosomes. Upon lysosomal blockage, we found an accumulation of autophagosomes in the perinuclear region and AGEs colocalized with these intracellular vesicles positive for the autophagosomal marker LC3 in lens and retina cells (Figure 1e and Figure S1 c‐e). Importantly, in both retinal pigment epithelial (RPE) and human lens epithelial cells (HLEC), we also found that AGEs colocalized with p62 in autophagic vesicles upon CQ treatment (Figure 1f and Figure S2 a‐c).

Overall, our data suggest a potential role for p62 in the targeting of endogenous AGEs to the autophagosomal compartment for degradation. Our study also shows that a deficit in the autophagic/lysosomal function results in accumulation of insoluble AGEs that could compromise cell viability.

### 2.2 Loss of the autophagic receptor p62 leads to glycation‐derived toxicity and AGEs accumulation *in vitro* and *in vivo*

**Figure 2:**
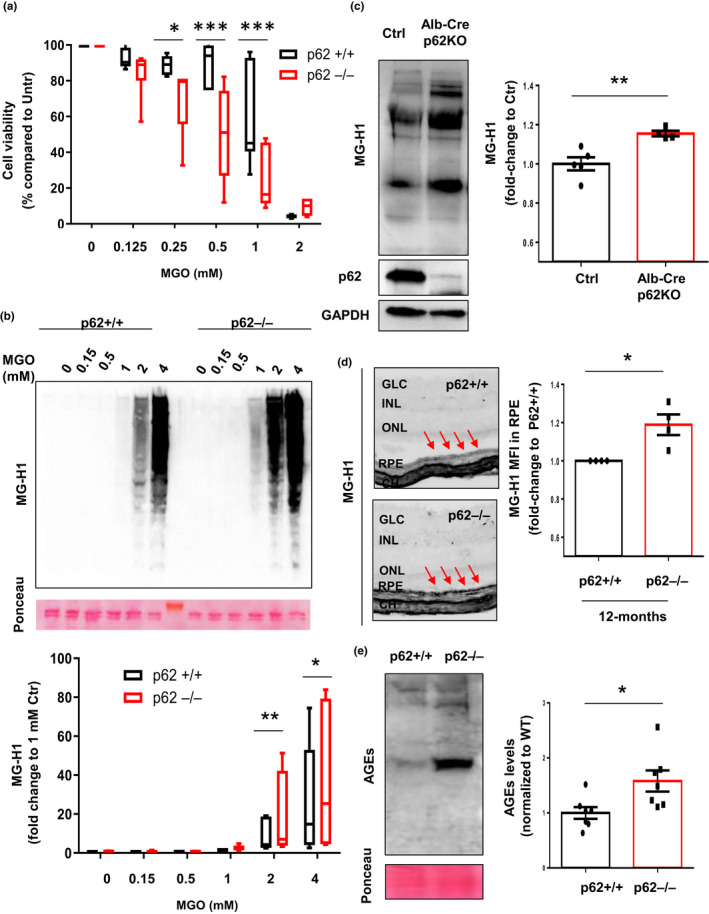
Lack of p62 leads to accumulation of AGEs *in vitro* and *in vivo*. (a) Viability of p62+/+ and p62−/− MEFs treated with the indicated concentrations of MGO for 24 h was measured by Cell‐Titer assay. Values are mean ±SEM (n = 6). We observed an interaction (*p* < 0.0001) between the MGO concentration and the genotype using two‐way ANOVA analysis. The differences between p62+/+ and p62−/− after the Sidak's multiple comparison test were significant for the 0.25, 0.5, and 1 mM doses of MGO (*p < 0.05 and ***p < 0.001). (b) Immunoblot for MG‐H1 in whole cellular extracts from WT MEFs (p62+/+) and MEFs lacking p62 (p62−/−). Representative immunoblot (*top*) and quantification of total levels of MG‐H1 relative to values in treated cells with 1 mM MGO (*bottom*). Values are mean ±SEM (n = 10). We observed an interaction (*p* = 0.03) between the MGO concentration and the genotype using two‐way ANOVA analysis. The differences between p62+/+ and p62−/− after the Sidak's multiple comparison test were significant for the 2 and 4 mM doses of MGO (*p < 0.05, **p < 0.01). (c) Immunoblot for MG‐H1 in liver tissues from WT and Alb‐Cre p62−/− mice. Representative immunoblot (*left*) and quantification of total levels of MG‐H1 relative to values in WT (*right*). Values are mean ±SEM (n = 5). (d) Representative immunohistochemistry for MG‐H1 in retinal tissues from 12‐month‐old p62+/+ and p62−/− mice (left) and quantification of MFI relative to values in p62+/+ (right). Arrows indicate the retinal pigment epithelial layer. Values are mean ±SEM (n = 5). Abbreviations: CH, choroid; RPE, retinal pigment epithelium; INL, inner nuclear layer; IPL, inner plexiform layer; ONL, outer nuclear layer; GCL, ganglion cell layer. (e) Immunoblot for AGEs in WT and p62−/− *C*. *elegans*. Representative immunoblot (*left*) and quantification of total levels of AGEs relative to values in WT (*right*). Values are mean ±SEM (n = 7). *p < 0.05, **p < 0.01 and ***p < 0.001

To assess the functional ramifications of p62‐dependent accumulation of AGEs, we tested if p62 plays a protective role against glycation‐derived toxicity. MGO, the primary biologic glycating metabolite of glucose reagent, leads to glycative stress and *in vitro* accumulation of AGEs (Uchiki et al., 2012). Mouse embryonic fibroblasts (MEFs) derived from wild‐type (WT) and p62 knockout animals were exposed to increasing concentrations of MGO for 24 hours (long‐term treatment). Concentrations of MGO ≤0.5 mM only caused a 10% decrease in cell viability in p62+/+ cells, while p62−/− cells displayed a fivefold greater susceptibility to glycation‐induced toxicity (Figure 2a). Even when exposed to 1 mM MGO for this extended treatment, 50% of p62+/+ cells remained viable, whereas only 20% of p62−/− cells survived. These data suggest that p62 confers viability protection in the face of glycative stress.

AGEs accumulation was observed following short‐term (2 hours) MGO exposure at concentrations above 1 mM MGO in MEFs lacking p62 more than in control MEFs (Figure 2b, compare lanes 12,13 versus 5,6). The difference between p62−/− and p62+/+ control cells was amplified at higher levels of MGO and at longer times of exposure (Figure 2b and Figure S2d) supporting the hypothesis that p62 has a crucial role in limiting homeostatic levels of AGEs.

The important role of p62 in the removal of AGEs was further explored *in vivo* in multiple mouse tissues (Figure 2c,d and Figure S3). In the whole body p62 knockout mouse, there was a 120% higher level of MG‐H1 in liver (Figure S3a,b). In the liver‐specific p62 knockout mouse, there was a 15% increase MG‐H1 (Figure 2c). The RPE is a major site of AGEs accumulation that is linked to AMD pathology (1, 27, 28). There was a 17% increase in AGEs in the RPE of 12‐month‐old whole body p62−/− mice (Figure 2d). We even observed a trend for accumulation of AGEs in the RPE of 3‐month‐old p62 knockout mice compared with age‐matched WT controls that were fed normal diets (Figure S3c,d). Further generalizing these observations, we also found 58% higher levels of AGEs in whole body *Caenorhabditis elegans* lacking the p62 ortholog, *sqst*‐*1*, compared with WT animals (Figure 2e), supporting a high degree of evolutionary conservation of this protective pathway. Together, these results lend further support to the hypothesis that p62 has a crucial role in limiting homeostatic levels of AGEs in various tissues in different organisms. Within each tissue or cell type, the group of AGEs that accumulates in the presence or absence of p62 have similar masses. This suggests that there is specificity to the glycation reactions within each cell or type of tissue.

**Figure 3:**
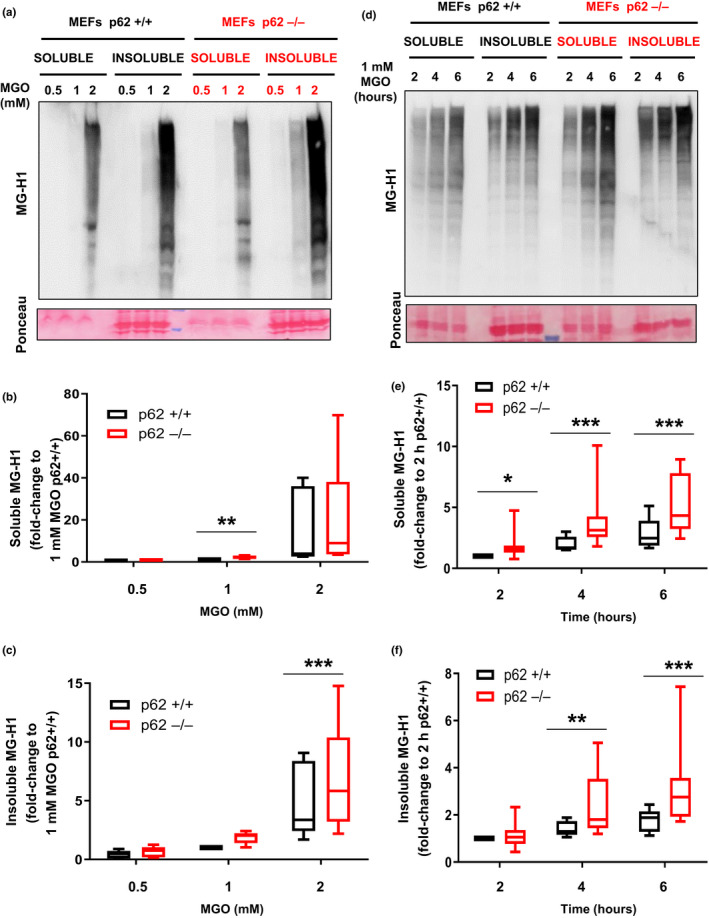
Absence of p62 leads to higher sensitivity against glycation‐derived burden. (a‐c) WT MEFs (p62+/+) and MEFs lacking p62 (p62−/−) were incubated with the indicated concentration of MGO for 2 hours, and lysates were separated into 1% Triton X‐100 soluble and insoluble fractions. (a) Soluble and insoluble fractions were immunoblotted for AGEs. Quantification of (b) soluble and (c) insoluble MG‐H1 in p62−/− MEF relative to values in 1 mM MGO treated p62+/+ cells. Values are mean ±SEM (n = 7). We observed an interaction (*p* < 0.01) between the MGO concentration and the genotype using two‐way ANOVA analysis only for the insoluble fraction (c). The differences between p62+/+ and p62−/− after the Sidak's multiple comparison test were significant for the 2 mM doses of MGO in the insoluble and 1 mM in the soluble fraction (**p < 0.01 and ***p < 0.001). (d‐f) Same cells were incubated with 1 mM of MGO for indicated times. (d) Representative immunoblot and quantification of (e) soluble and (f) insoluble MG‐H1 relative to values in 2 h treated p62+/+ cells. Values are mean ±SEM (n = 8). We observed an interaction (*p* < 0.01) between the time of MGO treatment and the genotype using two‐way ANOVA analysis for the soluble and insoluble fraction. *p < 0.05, **p < 0.01 and ***p < 0.001. The differences between p62+/+ and p62−/− after the Sidak's multiple comparison test were significant for the 2, 4, and 6 h of MGO in the soluble fraction and for 4 and 6 h of MGO in the insoluble fraction

Autophagy plays an essential role in clearance of insoluble cargoes. In order to further explore the role of p62, we asked if there is p62‐dependent partitioning of AGEs in Triton‐soluble and Triton‐insoluble fractions when cells are glycatively stressed. Concentrations above 1 mM MGO led to accumulation of soluble and insoluble AGEs in both WT and p62−/− MEFs, but AGEs content was higher in cells lacking p62 (Figure 3). AGEs accumulation was MGO concentration‐ and time‐dependent in both fractions. Importantly, at any specific concentration of MGO (compare Figure 3a lanes 9‐11 and 13‐15 versus 1‐3 and 5‐7, Figure 3b,c), or at any time, AGEs levels were higher in the insoluble fraction of the p62−/− cells versus the WT cells (compare Figure 3d, lanes 13‐15 versus lanes 5‐7, Figure 3d,e).

Together, these findings suggest that once glycation of a protein reaches a threshold, degradation of AGEs is not efficient, insolubilization or aggregation ensues, and this is associated with accelerated cytotoxicity under glycative stress.

### 2.3 p62‐dependent lysosomal targeting is compromised upon glycative stress

**Figure 4:**
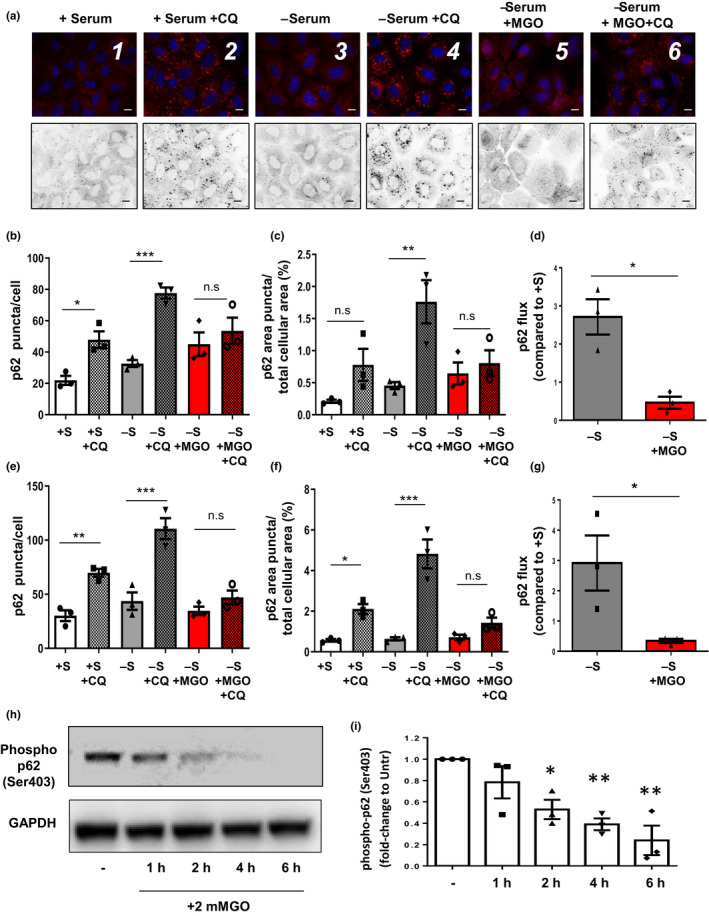
Glycative stress compromises p62 lysosomal targeting and Ser403 phosphorylation of p62. (a‐c) NRK cells were maintained for 2 h in complete medium (+S) or serum‐free medium (‐S) in the presence or absence of 2 mM MGO, 30 μM chloroquine (CQ) or both, fixed, and immunostained for endogenous p62. (a) Representative pictures are shown and quantifications of (b) average number of p62‐positive puncta per cell, (c) percentage of area occupied by p62‐positive puncta per total cellular area and (d) p62‐autophagic flux are shown. All values are mean ±SEM (n = 3, >30 cells per condition); * = *p* < 0.05; ** = *p* < 0.01; *** = *p* < 0.001. (e‐g) Similar parameters are shown from the analysis in HLECs incubated under the same conditions. Representative figures are showed in *SI Appendix* Figure S4. (h,i) U937 cells were incubated with 2 mM MGO for indicated times and phosphorylation of p62 at serine 403 was evaluated. (h) Representative immunoblot and (i) quantification of Ser403 phosphorylation of p62 relative to values in untreated cells. Values are mean ±SEM (n = 3). *p < 0.05 and **p < 0.01. For panels b, c, e, and f, one‐way ANOVA plus Sidak's multiple comparisons test were performed. For panel D and g, Student's t tests were performed. For panel i, one‐way ANOVA plus Dunnett's test was used to compare to control

Our observation of cytotoxicity and accumulation of AGEs upon glycative stress suggested that p62 and its function might also be victims of such stress, which, in turn, would then limit the targeting of AGEs to the autophagosome. Since p62 is trapped along with cargo in autophagic vesicles and degraded, accumulation of p62‐positive vesicles upon lysosomal blockage is an indicator of autophagic flux. Removal of serum from cells upregulates autophagy and was used to explore the fate of p62 in two different cell types highly sensitive to glycative stress and highly active for autophagy (Bejarano et al., 2012; Uchiki et al., 2012). As expected, inhibition of lysosomal function by CQ resulted in an increase in the number of p62‐positive puncta and of the subcellular area occupied by p62‐positive vesicles (Figure 4a, panels 4 versus 3, and 2 versus 1, b‐d). This was particularly obvious when autophagy was upregulated by serum starvation. However, when NRK cells were treated with MGO, the appearance of p62 puncta was markedly diminished, even in the presence of CQ, consistent with glycative stress limiting transfer of p62 to autophagosomes (Figure 4a, panel 6 versus 5, and 6 versus 4, b‐d). Similar data were obtained in HLECs (Figure S4 and Figure 4e‐g).

How might glycation interfere with transfer of p62 to autophagosomes? Phosphorylation of p62 at Serine 403 (S403) enhances its ability to recognize substrates and associate with autophagosomes (Matsumoto et al., 2011). We observed that glycative stress rapidly reduced the phosphorylated form of p62; phosphorylation levels fell to 50% after 2 hours of MGO treatment. (Figure 4h,i). Together, these findings indicate that targeting of p62 to lysosomes is compromised upon glycative stress.

**Figure 5:**
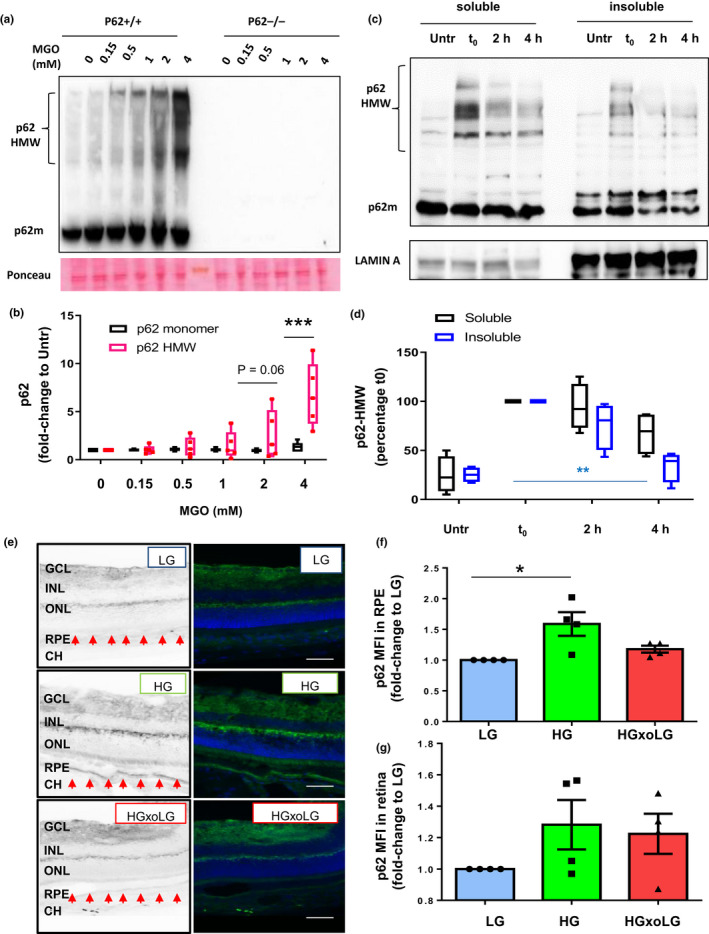
Accumulation of high molecular weight p62 upon glycative stress is reversible. (a,b) WT MEFs (p62+/+) and MEFs lacking p62 (p62−/−) were incubated with the indicated concentration of MGO for 2 hours, and whole cellular extracts were immunoblotted against p62. (a) Representative immunoblot and (b) quantification of p62 monomer and high molecular weight p62 (HMW‐p62) values relative to untreated cells are shown. Values are mean ±SEM (n = 5). We observed an interaction (*p* < 0.0001) between the MGO concentration and the HMW‐p62 using two‐way ANOVA analysis. The differences between HMW‐p62 and monomeric p62 after the Sidak's multiple comparison test were significant for the 4 mM doses of MGO (***p < 0.001). (c,d) ARPE‐19 cells were treated with 2 mM MGO for 2 hours followed by incubation in complete medium (no MGO) for either 2 or 4 hours. Cellular lysates were subjected to extraction with 1% Triton X‐100 and soluble and insoluble fractions were immunoblotted for the indicated proteins. (c) Representative immunoblot and (d) quantification of HMW‐p62 values relative to untreated cells are shown. Values are mean ±SEM (n = 5). Differences between t0 and insoluble p62 were significant for the 4 mM doses of MGO using one‐way ANOVA followed by Dunnett's multiple comparison test (**p < 0.01). (e,f) Retinal tissue sections from low‐glycemic (LG), high glycemic (HG), and crossover diet (HGxoLG) were analyzed immunohistochemically for p62. (e) Representative images of p62 immunostaining and mean intensity fluorescence in (f) the retinal pigment epithelial layer and (g) neuroretina relative to values in LG‐diet are shown. Values are mean ±SEM (n = 4). Abbreviations: CH, choroid; RPE, retinal pigment epithelium; INL, inner nuclear layer; IPL, inner plexiform layer; ONL, outer nuclear layer; GCL, ganglion cell layer. *p* < 0.05 in one‐way ANOVA followed by Dunnett's multiple comparison test

In order to determine if p62 suffers the same fate as other proteins upon glycative stress, including crosslinking and aggregation, we specifically monitored p62 fate. Upon short incubation of MEFs with MGO, p62 accumulated in high molecular weight forms (HMW‐p62), consistent with not only stress but also compromised p62 autophagic function (Aki et al., 2019; Donohue et al., 2014; Zhang et al., 2017). We observed that increasing concentrations of MGO led to accumulation of HMW‐p62 (Figure 5a‐b). Of note, levels of HMW‐p62 fell rapidly when stress was removed (Figure 5c‐d), consistent with the glycation‐induced aggregation of p62 being reversible and/or degradation of AGEs being enhanced upon removal of the stress (Aki et al., 2019). The data also indicate that the cells remained viable during this short‐term treatment.

These observations also reflect *in vivo* experience. AGEs accumulate in the RPE layer and are involved in the AMD pathogenesis that is observed in mice that consume higher glycemic index (GI) diets and that model human AMD (Rowan et al., 2017). p62 levels increased 59% in the RPE of aged mice fed a high‐GI diet (Figure 5e‐f). Statistically significant differences were not observed in neuroretina between high‐GI diet and low GI diet (Figure 5g), suggesting that the RPE is more sensitive than other ocular tissues to glycative stress and corroborating observations that the RPE is the nidus of AMD pathology (Rowan et al., 2017; Weikel et al., 2012). As in the cell culture experiments, switching from the high GI diet to the low GI diet lowered p62 to basal homeostatic levels (Figure 5e‐f), validating the cell culture observations shown in Figure 5a‐c, and extending this to an important *in vivo* model of AMD. This is of particular interest, since removal of the glycemic stress has been reported to reverse or stop accumulation of retina pathology that is etiologic of AMD (Rowan et al., 2017).

### 2.4 Enhanced autophagy protects against glycative damage *in vitro* and *in vivo*

**Figure 6:**
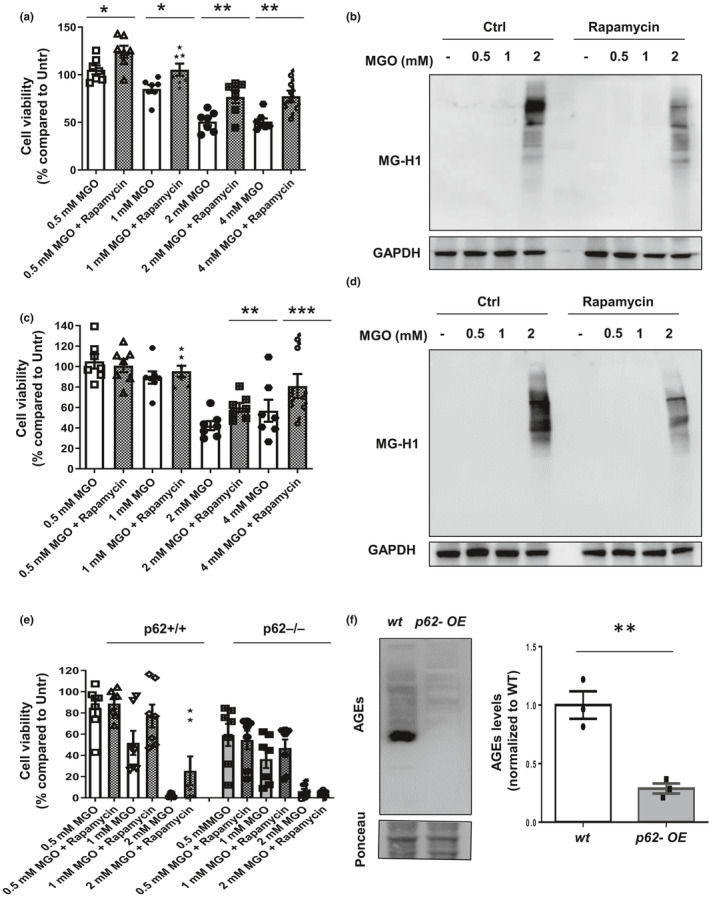
Enhanced autophagy protects against glycative damage by reducing AGEs accumulation. (a, b) ARPE‐19 cells were treated with the indicated concentrations of MGO in the absence or presence of 1 µM rapamycin. (a) Cell viability was measured by Cell‐Titer assay. Values are mean ±SEM (n = 7). We observed significant effects of both the MGO concentration and the rapamycin using two‐way ANOVA analysis (*p* < .00001). The differences after rapamycin treatment were significant for all the doses of MGO after the Sidak's multiple comparison test (*p < 0.05, **p < 0.01). (b) Immunoblot against MG‐H1 is shown. (c, d) HLECs cells were treated under the same conditions. (c) Cell viability, and (d) immunoblot against MG‐H1 are shown. Values are mean ±SEM (n = 7). We observed interaction between the MGO concentration and rapamycin using two‐way ANOVA analysis (*p* = 0.0035). The protective effect of rapamycin treatment on cell survival was significant for the 2 and 4 mM doses of MGO after the Sidak's multiple comparison test (**p < 0.01 and ***p < 0.001). (e) WT MEFs (p62+/+) and MEFs lacking p62 (p62−/−) were incubated with the indicated concentrations of MGO in the absence or presence of 1 µM rapamycin and cell viability was analyzed. Values are mean ±SEM (n = 7). We analyzed the effects of p62 genotype, MGO dose, and rapamycin using 3‐way ANOVA matching by MGO dose and rapamycin. The three factors have significant effect: p62 genotype (*p < 0.05), MGO dose (***p < 0.001), and rapamycin (**p < 0.01). The only significant interaction was between rapamycin and p62 genotype (*p* = 0.0077), because it has protective effect only in p62+/+ MEFs. (F) Immunoblot against AGEs in WT and p62‐overexpressing *C*. *elegans*. Representative immunoblot (left) and quantification of total levels of MG‐H1 relative to values in WT (right) Values are mean ±SEM (n = 3). *p < 0.05, **p < 0.01 and ***p < 0.001

Next, we tested if enhancement of autophagy might promote cell survival upon glycative stress. Autophagy was pharmacologically enhanced using rapamycin. This binds to and inhibits mTOR, mimicking nutrient depletion and stimulating autophagy. As with p62+/+ versus p62−/− MEFs and long exposure (24 hours) to MGO (Figure 2), we observed increased viability in RPE cells exposed to rapamycin compared to control cells at MGO concentrations greater than 1 mM (*p* = 0.03 for 0.5 mM MGO, *p* = 0.02 for 1 mM MGO, *p* = 0.002 for 2 mM MGO, and *p* = 0.002 for 4 mM MGO) (Figure 6a). This was associated with decreased AGEs (Figure 6b, compare lanes 9 versus 4). The results were replicated in HLECs (Figure 6c,d).

Of note, this protective effect of autophagy enhancement was observed only in cells replete with p62. That is, we observed that rapamycin was protective in control cells, but not in MEFs lacking p62 (Figure 6e).

The protection by p62‐dependent autophagy against glycative stress was then tested *in vivo*. Overexpressing the p62 ortholog SQST‐1 in *C*. *elegans*, which induces autophagy (Kumsta et al., 2019), reduced the levels of endogenous AGEs 70% (Figure 6f). Of note, accumulation of AGEs was greatest in *C*. *elegans* from which p62 was deleted and least in *C*. *elegans* in which p62 was overexpressed (Figure S2e). These findings further support a conserved and critical role of this autophagic receptor in the maintenance of non‐toxic homeostatic AGEs levels.

## 3 DISCUSSION

The accumulation of AGEs during physiological aging, particularly upon consumption of high glycemic diets, is etiologically associated with multiple age‐related disorders including CVD, diabetes, and AMD, but the mechanisms underlying these associations have not been well explored (Bejarano & Taylor, 2019; Chaudhuri et al., 2018; Moldogazieva & Mokhosoev, 2019; Vicente Miranda et al., 2016). In this work, we observe that AGEs accumulate due to impaired recruitment of autophagic cargo in a wide range of organisms, tissues, and cells, both *in vitro* and *in vivo*. Specifically, this work reveals that the autophagy recruitment factor p62 plays a key role in moving AGEs to lysosomal compartments for their degradation. Compromised p62 capacity leaves cells and tissues vulnerable. This can be reversed when p62 is augmented, revealing many opportunities to decrease glycative stress and its pathologic sequelae.

Emerging evidence regarding detrimental effects of AGEs on organismal homeostasis and health, particularly with advancing age, prompted us to explore strategies to delay AGEs accumulation. Specifically, we were motivated to explore proteolytic capacities that might be harnessed to limit AGEs accumulation because: 1) research to chemically inhibit the formation of these glycotoxins has not yielded clinically useful drugs in human trials (Nenna et al., 2015), 2) AGEs accumulation and a decline in autophagic activity are characteristic features of aging, 3) genetic strategies that enhance autophagy or diminish AGEs have been associated with extended lifespan in model organisms and abrogate some age‐related pathologies (Fernandez et al., 2018; Golegaonkar et al., 2015; Lapierre et al., 2013; Morcos et al., 2008; Schlotterer et al., 2009; Simonsen et al., 2008), and 4) conversely, lack of autophagy or increased glycative stress trigger degenerative changes that resemble aging‐associated features such as accumulation of dysfunctional organelles and ubiquitin aggregates (Rubinsztein et al., 2011; Uchiki et al., 2012).

AGEs deposition was observed in rat brains and mouse retinas, as well as in human lens and RPE cells, when animals consumed higher glycemic diets or upon prolonged or enhanced glycative stress (Rowan et al., 2017; Uchiki et al., 2012; Weikel et al., 2012). These effects are exacerbated upon aging. We note here more high mass AGEs in the insoluble fraction at a given concentration of MGO, suggesting that once glycation of a protein ensues, it continues to react locally until insoluble moieties are formed. This is corroborated by the transition to accumulation of higher mass AGEs in all fractions with increasing time (Figure 3). Accumulation of AGEs was also obvious when either the UPS or autophagy was inhibited (Figure 1) (Uchiki et al., 2012). Since AGEs accumulation was reversed in diverse cells when autophagy was stimulated, and this conferred viability (Figure 6), we hypothesized that specific mechanisms of autophagy are salutary, at least in part, by providing a means to limit AGEs accumulation (Takahashi et al., 2017; Uchiki et al., 2012).

We focused on p62, a major carrier of autophagic cargoes to lysosomes, and itself an autophagic target, because initial experiments indicated that inhibition of autophagy resulted in accumulation of this carrier along with AGEs (Figure 1b, Figure S1a). Robust evidence that p62 is a major mediator of autophagic degradation of AGEs shown here include the following: 1) markedly diminished levels of AGEs in *C*. *elegans in vivo* in which p62 was overexpressed (Figure 6f), and, similarly, markedly diminished levels of AGEs *in vivo* in mice and *C*. *elegans* when p62 was present as opposed to being knocked out (Figure 2b‐e, S3). 2) Enhanced cell viability in the face of glycative stress when p62 was present (Figure 2a). 3) Only when p62 is present does pharmacological upregulation of autophagy increase cell survival in the face of glycative stress (Figure 6e). 4) elevated AGEs, ubiquitin and p62 when autophagy is inhibited (Figure 1b, Figure S1a, Figure S2a‐c). 5) We observe less accumulation of AGEs in metabolically diverse tissues and cells that express p62 as opposed to those that do not, particularly in the insoluble fractions (Figures 2b‐e, 3a‐f, Figure S3a). 6) More accumulation of p62 in aged tissues, in cells when autophagy is inhibited pharmacologically or by glycative stress, or in tissues from animals that consumed a higher glycemia diet (Figures 1a,b, 4, 5b,e,f; Figures. S2a‐c, S4). These observations are consistent with prior findings that treatment with rapamycin, an mTOR inhibitor and, consequently, an autophagy activator, and p62 overexpression have been shown to increase lifespan in different organisms and preclude age‐related dysfunction (Aparicio et al., 2020; Flynn et al., 2013; Kumsta et al., 2019) and limits pathological deposits of β‐amyloid and tau proteins (Caccamo et al., 2010; Ozcelik et al., 2013; Spilman et al., 2010).

Additional evidence for a crucial role for p62 in AGEs‐related detoxification was gleaned from observations that the activity of p62 was inversely related to glycative stress (Figure 4h,i) and cells confronted with glycative stress accumulate far more high mass SDS‐resistant cross‐linked p62 and less p62 in autophagosomes (Figure 5a; 4a and S4). Like large ubiquitin aggregates, these are probably dysfunctional (Taylor, 2012). p62 targeting to lysosomal compartments was lower in glycatively stressed cells (Figure 4, Figure S4). Biologic relevance of a p62 response to glycative damage is evident in the enhanced accumulation of high mass p62 aggregates in the RPE of mice reared on high glycemic index diets and in conditions with impaired autophagy (Aki et al., 2019; Donohue et al., 2014; Zhang et al., 2017). The diminution of p62 when the animals were returned to a lower glycemia diet indicates salutary reversibility (Figure 5e). These findings corroborate prior observations that hallmarks of retinal damage were also arrested or reversed when animals were returned to lower glycemic diets after having consumed high glycemic diets for over 6 months. This also shows that the stress, which reduced p62 function, can be reversed. This opens up promising modalities to treat diseases such as AMD by easily achievable, cost‐effective, and dietary intervention(Rowan et al., 2017; Taylor, 2012).

Our identification of p62 as a biologically conserved mediator of AGEs clearance associated with survival and disease suggests that it would be profitable to explore drug targets to enhance its activity or stability (Pankiv et al., 2007). Since there are other mammalian autophagic receptors, such as NBR1, NDP52, TAX1BP, and OPTN, future studies will interrogate their function in removal of AGEs and as targets for pharmacotherapy (Birgisdottir et al., 2013).

In sum, we propose a model in which basal autophagic activity contributes to the clearance of endogenous AGEs that are formed through the metabolism of sugars and, additionally, that p62‐dependent autophagy participates in safeguarding cells and tissues in response to AGEs overload (Figure S5). Furthermore, at levels of glycative stress that leave p62 functional, it is a major executor of clearance of AGEs. At elevated levels of glycative damage, p62 is rendered dysfunctional. Collectively, our findings suggest that combined glycative stress and defective p62‐dependent autophagy contribute to the loss of proteostasis with age and the cytotoxic accumulation of damaged proteins and cell death, all hallmarks of aging (Lopez‐Otin et al., 2013; Moldogazieva & Mokhosoev, 2019). Taken together with experiments that show it is possible to accelerate clearance of AGEs and retain viability, these data suggest p62 as a new target for interventions to mitigate damage due to accumulation of AGEs. The relationship between autophagy and glycative stress‐induced toxicity is conserved across multiple cell types, and in diverse species from *C*. *elegans* to mammals. It is notable that cell or tissue tolerances to glycative damage differ, as indicated by the observation of accumulation of p62 and AGEs in RPE in high glycemia fed mice, but not in neuroretina (Figure 6f,g) (Rowan et al., 2018). The specific sensitivity of the RPE as a site of accumulation of AGEs is consistent with the RPE being the nidus of AMD. Since glycation has been shown to affect and enhance aggregation patterns of cataractogenic lens as well as neurotoxic α‐synuclein and tau proteins associated with Parkinson's and Alzheimer's disease, respectively, these findings suggest that p62‐dependent autophagy induction may be salutary with regard to these widely prevalent pathologies as well (Emendato et al., 2018; Liu et al., 2016; Vicente Miranda et al., 2016).

## 4 EXPERIMENTAL PROCEDURES

### 4.1 Animal husbandry

C57BL/6 J p62 knockout mice (allele designation is *Sqstm1^tm1Keta^*) and liver‐specific p62 knockout were donated by Dr. Komatsu Masaaki (Juntendo University, Japan), and WT mice were fed regular chow diet for either three or twelve months. Details regarding the high‐GI (HG) or low GI (LG) diet‐fed mice can be found in (Rowan et al., 2017). In brief, C57BL/6 J WT mice were purchased from Jackson Laboratories and fed standard chow until 12 months of age. Then, the mice were placed on either HG or LG diets and were pair fed. At 18 months of age (6 months after the diets were commenced), half of the HG mice were changed over to the LG diet (HGxoLG). The diets were isocaloric and contained all essential micronutrients and identical macronutrient compositions except that HG starch was 100% amylopectin (Amioca starch; Ingredion, Inc.), whereas the LG starch was composed of 70% amylose/30% amylopectin (HYLON VII starch; Ingredion Incorporated) (Rowan et al., 2017; Uchiki et al., 2012; Weikel et al., 2012). All diets consisted of 65% carbohydrate, 21% protein, and 14% fat and were formulated by Bio‐Serv. After sacrifice, the tissues were collected either in fixative or flash‐frozen in liquid nitrogen. Tissues were embedded in OCT for immunohistochemistry. 10 μm cryosections were obtained, dried overnight, and stored at −80 °C until staining. This study was conducted under an animal study protocol approved by the Institutional Animal Care and Use Committee at Tufts University.

### 4.2 Cell culture and treatments

Human lens epithelial cells (HLEC, line SRA 01/04) were a gift from Dr. Venkat Reddy, (Kellogg Eye Center, University of Michigan). Human retinal pigment epithelial cells (ARPE19), normal rat kidney cells (NRK), and U937 were from the American Type Culture Collection. Mouse embryonic fibroblasts (MEFs) from mice WT (p62+/+) and null for p62 (p62−/−) were a gift from Dr. Masaaki Komatsu (Juntendo University, Japan). All cells were cultured in DMEM or RPMI 1640 medium (GIBCO) containing 10% FBS, 50 µg/ml penicillin, and 50 µg/ml streptomycin at 37°C with 5% CO2 and periodically tested for mycoplasma contamination using a DNA staining protocol with Hoechst 33258 dye. Serum removal was performed by thoroughly washing the cells with PBS (Lonza) and placing them in serum‐free medium. Where indicated, lysosomal proteolysis was inhibited by addition of 30 μM CQ, enhanced autophagy was achieved with 1 µM Rapamycin, and glycative stress was induced by addition of MGO. All the studies with MGO were performed at cell density of 90% confluency, as previously described (Uchiki et al., 2012). The fluorometric CellTiter‐Blue Assay from Promega was used for assessing cell viability.

### 4.3 Antibodies and chemicals

The following primary antibodies were used in the study: p62 (#PM045) from MBL International; LC3 (#NB100‐2220) from Novus Biologicals; p62 (#ab56416), Lamin A (#ab58528) from Abcam; GAPDH (#G9545) from Sigma; phospho‐SQSTM1/p62 (Ser403) (#14354) from Cell Signaling Technology; MG‐H1 (#STA‐011) from Cell Biolabs. The antibody against MG‐H1 used in Main Figures 1e,f, 2d, Suppl Figure 1c‐f, Suppl Figure 2a‐d, Suppl Figure 3c, and in immunoblots of mouse samples in Main Figure 2c and Figure S3a was a kind gift from Drs. Michael Brownlee and Xue‐Liang Du (Albert Einstein College of Medicine, New York, USA). An anti‐AGE #ab23722 from Abcam was used in *C*.*elegans* samples in Main Figures 2e and 6f. For the rest of the manuscript the commercial antibody #STA‐011 was used for quantitative purposes. The antibody rabbit polyclonal antibody against ubiquitin was produced in this laboratory (Shang & Taylor, 1995). Texas Red‐ and FITC‐conjugated secondary antibodies were purchased from Jackson ImmunoResearch Laboratories. Secondary antibodies against mouse and rabbit conjugated to HRP were purchased from Vector Laboratories. Methylglyoxal (MGO) and chloroquine (CQ) were obtained from Sigma‐Aldrich. Penicillin‐streptomycin solution and FBS, non‐essential amino acids, and sodium pyruvate were from GIBCO and protease inhibitor cocktail from Roche. Rapamycin was purchased from LC Laboratories, USA.

### 4.4 Detergent solubility assay and immunoblot analysis

Cells were rinsed with phosphate‐buffered saline (PBS) at 4°C, collected and centrifuged at 1,000 × g for 5 min. Total whole cellular extracts were prepared by resuspending the cellular pellets in PBS with protease inhibitors, sonicated, and the amount of protein in the samples was estimated using BCA Protein Assay Kit (Pierce). The detergent solubility assay with 1% Triton X‐100 was performed as described previously (Bejarano et al., 2012). Briefly, soluble fractions were prepared by solubilization in RIPA buffer (1% Triton X‐100, 1% sodium deoxycholate, 0.1% SDS, 0.15 M NaCl and 0.01 M sodium phosphate, at pH 7.2) containing protease inhibitors. Cellular pellets were resuspended and incubated on ice for 20 min. The soluble fractions were recovered as the supernatants after centrifugation at 13,000 × g for 30 min. The detergent‐insoluble pellets were washed twice in PBS, resuspended in Laemmli buffer and sonicated. Laemmli buffer was also added to the Triton X‐100–soluble fractions, and the samples were denatured at 100°C for 5 min before SDS‐PAGE and immunoblotting. For *C*. *elegans* samples, *C*. *elegans* strains used were WT (N2) animals, p62−/− animals, that is, *sqst*‐*1(syb764)* (MAH914), and p62‐overexpressing animals, that is, WT animals expressing *sqIs35[sqst*‐*1p*::*sqst*‐*1*::*gfp *+* unc*‐*122p*::*rfp]* (MAH349) (Kumsta et al., 2019). Animals were maintained and cultured under standard conditions at 20°C and age‐synchronized using hypochlorite solution. On day 1 of adulthood, animals were washed off the growth media plates and 200 μl of densely packed worms were mixed with an equal volume of Zirconia beads and lysed in a Fastprep cell disrupter. The protein concentration was determined, and 50 μg of total protein was loaded on onto 4‐12% Bis‐Tris protein gel (Thermo Fisher Scientific) for separation and transferred to a PVDF membrane (Millipore) for immunoblotting. Proteins were identified using specific antibodies followed by visualization using peroxidase‐conjugated secondary antibodies. Membranes were developed using MultiImage II Alpha Imager (Alpha Innotech).

### 4.5 Immunostaining and image analysis

Indirect immunofluorescence was performed following conventional procedures, as previously described (Bejarano et al., 2012; Rowan et al., 2017). Briefly, cells were grown on coverslips, fixed for 10 min in either ice‐cold methanol or 4% formaldehyde in PBS, blocked and permeabilized 10 min with PBS containing 0.5% BSA, 0.01% Triton X‐100 and then incubated with the primary antibody followed by corresponding Alexa 488‐ or Texas Red‐conjugated secondary antibodies. After immunostaining, cells were rinsed with PBS and mounted for microscopy using Fluoromount‐G containing DAPI to highlight the nuclei. Immunohistochemistry was carried out as previously described (Bejarano et al., 2012; Rowan et al., 2017; Bejarano et al., 2018). Bleaching on retina sections was performed by incubating the sections in 10% H_2_O_2_ at 65⁰C for 1.5 hours. After staining, slides were mounted in Prolong Gold Antifade with DAPI (Molecular Probes). Images were collected in a Axiovert 200 fluorescence microscope (Carl Zeiss, Jena, Germany) equipped with 20× and 100× objectives. For analysis of p62 activity, a quantitative analysis was performed according to the guidelines for the use and interpretation of assays for monitoring autophagy (Klionsky et al., 2016). The number of fluorescent puncta and area occupied by p62‐positive puncta per cell was calculated using ImageJ. Subtraction between densitometric values in presence of CQ and in absence of the lysosomal inhibitor represents the p62‐autophagic flux, the amount of p62 degraded in the lysosomes. For the analysis of immunological sections, mean fluorescence intensity (MFI) was calculated and normalized to WT samples (Rowan et al., 2017).

### 4.6 Image and statistical analysis

Densitometric quantification of immunoblots was performed in unsaturated images using ImageJ (NIH). All Western blot data were normalized to protein loading control (GAPDH or Ponceau), and the values are expressed as relative levels or percent change compared with untreated cells. In order to quantify levels of AGEs in immunoblot, the whole lane for the MG‐H1 was analyzed and densitometric values were calculated. For quantitative analysis of immunofluorescence, single images were taken at the section of the maximum nucleus diameter and all quantifications were done blindly (Bejarano et al., 2014). The number and area occupied by the fluorescent particles were determined using the Analyze Particles function of ImageJ (NIH) after applying a fixed threshold to all images. All numerical results are reported as the mean ±SEM from a minimum of three independent experiments. In all instances “n” refers to individual experiments, indicated in the corresponding figure legends. GraphPad InStat software (GraphPad) was used for analysis of statistical significance. One‐way ANOVA followed by Dunnett's multiple comparison test was used when values were compared to untreated controls. Two‐way ANOVA followed by the Sidak's multiple comparison test was used when we compared selected pairs of means. Three‐way ANOVA followed by Tukey's multiple comparison test was carried out to analyze the effect of genotype, MGO dose and rapamycin in Figure 6e. Two‐tailed Student's t test was used to evaluate single comparisons between different experimental groups. Differences were considered statistically significant for a value of *p* < 0.05 and denoted by an asterisk in the graph.

## CONFLICT OF INTEREST

The authors declare no conflict of interest.

## AUTHORS’ CONTRIBUTIONS

G.A, O.O, C.R, J.V., M.W., K.D., and W.Y provided technical assistance and performed biochemical experiments; S.F and S.R carried out quantitative immunohistochemistry and revised the written manuscript; E.W and J.A helped with experimental design and contributed to revision of the paper; C.K and M.H provided *C*. *elegans* samples; S.K and K.M provided p62 knockout mice along with Alb‐Cre liver tissues; X.D and M.B provided primary antibody against MG‐H1; P.D, H.D, and D.R provided samples from hippocampi of young and old rats; E.B and A.T. designed the experiments, analyzed the data, coordinated the study, and wrote the paper.

## Data Availability

Data sharing not applicable to this article as no datasets were generated or analyzed during the current study.

## References

[acel13257-bib-0001] Aki, T. , Unuma, K. , Noritake, K. , Hirayama, N. , Funakoshi, T. , & Uemura, K. (2019). Formation of high molecular weight p62 by CORM‐3. PLoS One, 14(1), e0210474 10.1371/journal.pone.0210474 30620762 PMC6324786

[acel13257-bib-0002] Aparicio, R. , Hansen, M. , Walker, D. W. , & Kumsta, C. (2020). The selective autophagy receptor SQSTM1/p62 improves lifespan and proteostasis in an evolutionarily conserved manner. Autophagy, 16(4), 772‐774. 10.1080/15548627.2020.1725404 32041473 PMC7138197

[acel13257-bib-0003] Bejarano, E. , Girao, H. , Yuste, A. , Patel, B. , Marques, C. , Spray, D. C. , Pereira, P. , & Cuervo, A. M. (2012). Autophagy modulates dynamics of connexins at the plasma membrane in a ubiquitin‐dependent manner. Molecular Biology of the Cell, 23(11), 2156‐2169. 10.1091/mbc.E11-10-0844 22496425 PMC3364179

[acel13257-bib-0004] Bejarano, E. , Murray, J. W. , Wang, X. , Pampliega, O. , Yin, D. , Patel, B. , Yuste, A. , Wolkoff, A. W. , & Cuervo, A. M. (2018). Defective recruitment of motor proteins to autophagic compartments contributes to autophagic failure in aging. Aging Cell, 17(4), e12777 10.1111/acel.12777 29845728 PMC6052466

[acel13257-bib-0005] Bejarano, E. , & Taylor, A. (2019). Too sweet: Problems of protein glycation in the eye. Experimental Eye Research, 178, 255‐262. 10.1016/j.exer.2018.08.017 30145354 PMC8351608

[acel13257-bib-0006] Bejarano, E. , Yuste, A. , Patel, B. , Stout, R. F. Jr , Spray, D. C. , & Cuervo, A. M. (2014). Connexins modulate autophagosome biogenesis. Nature Cell Biology, 16(5), 401‐414. 10.1038/ncb2934 24705551 PMC4008708

[acel13257-bib-0007] Birgisdottir, A. B. , Lamark, T. , & Johansen, T. (2013). The LIR motif ‐ crucial for selective autophagy. Journal of Cell Science, 126(Pt 15), 3237‐3247. 10.1242/jcs.126128 23908376

[acel13257-bib-0008] Caccamo, A. , Majumder, S. , Richardson, A. , Strong, R. , & Oddo, S. (2010). Molecular interplay between mammalian target of rapamycin (mTOR), amyloid‐beta, and Tau: effects on cognitive impairments. Journal of Biological Chemistry, 285(17), 13107‐13120. 10.1074/jbc.M110.100420 20178983 PMC2857107

[acel13257-bib-0009] Chaudhuri, J. , Bains, Y. , Guha, S. , Kahn, A. , Hall, D. , Bose, N. , Gugliucci, A. , & Kapahi, P. (2018). The role of advanced glycation end products in aging and metabolic diseases: bridging association and causality. Cell Metabolism, 28(3), 337‐352. 10.1016/j.cmet.2018.08.014 30184484 PMC6355252

[acel13257-bib-0010] Donohue, E. , Balgi, A. D. , Komatsu, M. , & Roberge, M. (2014). Induction of Covalently Crosslinked p62 Oligomers with Reduced Binding to Polyubiquitinated Proteins by the Autophagy Inhibitor Verteporfin. PLoS One, 9(12), e114964 10.1371/journal.pone.0114964 25494214 PMC4262463

[acel13257-bib-0011] Emendato, A. , Milordini, G. , Zacco, E. , Sicorello, A. , Dal Piaz, F. , Guerrini, R. , & Pastore, A. (2018). Glycation affects fibril formation of Abeta peptides. Journal of Biological Chemistry, 293(34), 13100‐13111. 10.1074/jbc.RA118.002275 29959224 PMC6109928

[acel13257-bib-0012] Fernández, Á. F. , Sebti, S. , Wei, Y. , Zou, Z. , Shi, M. , McMillan, K. L. , He, C. , Ting, T. , Liu, Y. , Chiang, W.‐C. , Marciano, D. K. , Schiattarella, G. G. , Bhagat, G. , Moe, O. W. , Hu, M. C. , & Levine, B. (2018). Disruption of the beclin 1‐BCL2 autophagy regulatory complex promotes longevity in mice. Nature, 558(7708), 136‐140. 10.1038/s41586-018-0162-7 29849149 PMC5992097

[acel13257-bib-0013] Flynn, J. M. , O'Leary, M. N. , Zambataro, C. A. , Academia, E. C. , Presley, M. P. , Garrett, B. J. , Zykovich, A. , Mooney, S. D. , Strong, R. , Rosen, C. J. , Kapahi, P. , Nelson, M. D. , Kennedy, B. K. , & Melov, S. (2013). Late‐life rapamycin treatment reverses age‐related heart dysfunction. Aging Cell, 12(5), 851‐862. 10.1111/acel.12109 23734717 PMC4098908

[acel13257-bib-0014] Gavilan, E. , Pintado, C. , Gavilan, M. P. , Daza, P. , Sanchez‐Aguayo, I. , Castano, A. , & Ruano, D. (2015). Age‐related dysfunctions of the autophagy lysosomal pathway in hippocampal pyramidal neurons under proteasome stress. Neurobiology of Aging, 36(5), 1953‐1963. 10.1016/j.neurobiolaging.2015.02.025 25817083

[acel13257-bib-0015] Golegaonkar, S. , Tabrez, S. S. , Pandit, A. , Sethurathinam, S. , Jagadeeshaprasad, M. G. , Bansode, S. , & Mukhopadhyay, A. (2015). Rifampicin reduces advanced glycation end products and activates DAF‐16 to increase lifespan in Caenorhabditis elegans. Aging Cell, 14(3), 463‐473. 10.1111/acel.12327 25720500 PMC4406675

[acel13257-bib-0016] Ji, C. H. , & Kwon, Y. T. (2017). Crosstalk and Interplay between the Ubiquitin‐Proteasome System and Autophagy. Molecules and Cells, 40(7), 441‐449. 10.14348/molcells.2017.0115 28743182 PMC5547213

[acel13257-bib-0017] Johansen, T. , & Lamark, T. (2011). Selective autophagy mediated by autophagic adapter proteins. Autophagy, 7(3), 279‐296. 10.4161/auto.7.3.14487 21189453 PMC3060413

[acel13257-bib-0018] Kazi, R. S. , Banarjee, R. M. , Deshmukh, A. B. , Patil, G. V. , Jagadeeshaprasad, M. G. , & Kulkarni, M. J. (2017). Glycation inhibitors extend yeast chronological lifespan by reducing advanced glycation end products and by back regulation of proteins involved in mitochondrial respiration. Journal of Proteomics, 156, 104‐112. 10.1016/j.jprot.2017.01.015 28132874

[acel13257-bib-0019] Klionsky, D. J. , Abdelmohsen, K. , Abe, A. , Abedin, M. J. , Abeliovich, H. , Acevedo Arozena, A. , Adachi, H. , Adams, C. M. , Adams, P. D. , Adeli, K. , Adhihetty, P. J. , Adler, S. G. , Agam, G. , Agarwal, R. , Aghi, M. K. , Agnello, M. , Agostinis, P. , Aguilar, P. V. , Aguirre‐Ghiso, J. , … Zughaier, S. M. (2016). Guidelines for the use and interpretation of assays for monitoring autophagy. Autophagy, 12(1), 1‐222. 10.1080/15548627.2015.1100356 26799652 PMC4835977

[acel13257-bib-0020] Komatsu, M. , Waguri, S. , Koike, M. , Sou, Y.‐S. , Ueno, T. , Hara, T. , Mizushima, N. , Iwata, J.‐I. , Ezaki, J. , Murata, S. , Hamazaki, J. , Nishito, Y. , Iemura, S.‐I. , Natsume, T. , Yanagawa, T. , Uwayama, J. , Warabi, E. , Yoshida, H. , Ishii, T. , … Tanaka, K. (2007). Homeostatic levels of p62 control cytoplasmic inclusion body formation in autophagy‐deficient mice. Cell, 131(6), 1149‐1163. 10.1016/j.cell.2007.10.035 18083104

[acel13257-bib-0021] Kumsta, C. , Chang, J. T. , Lee, R. , Tan, E. P. , Yang, Y. , Loureiro, R. , Choy, E. H. , Lim, S. H. Y. , Saez, I. , Springhorn, A. , Hoppe, T. , Vilchez, D. , & Hansen, M. (2019). The autophagy receptor p62/SQST‐1 promotes proteostasis and longevity in C. elegans by inducing autophagy. Nature Communications, 10(1), 5648 10.1038/s41467-019-13540-4 PMC6906454 31827090

[acel13257-bib-0022] Lapierre, L. R. , De Magalhaes Filho, C. D. , McQuary, P. R. , Chu, C.‐C. , Visvikis, O. , Chang, J. T. , Gelino, S. , Ong, B. , Davis, A. E. , Irazoqui, J. E. , Dillin, A. , & Hansen, M. (2013). The TFEB orthologue HLH‐30 regulates autophagy and modulates longevity in Caenorhabditis elegans. Nature Communications, 4, 2267 10.1038/ncomms3267 PMC3866206 23925298

[acel13257-bib-0023] Liu, K. , Liu, Y. , Li, L. , Qin, P. , Iqbal, J. , Deng, Y. , & Qing, H. (2016). Glycation alter the process of Tau phosphorylation to change Tau isoforms aggregation property. Biochimica Et Biophysica Acta, 1862(2), 192‐201. 10.1016/j.bbadis.2015.12.002 26655600

[acel13257-bib-0024] Lopez‐Otin, C. , Blasco, M. A. , Partridge, L. , Serrano, M. , & Kroemer, G. (2013). The hallmarks of aging. Cell, 153(6), 1194‐1217. 10.1016/j.cell.2013.05.039 23746838 PMC3836174

[acel13257-bib-0025] Matsumoto, G. , Wada, K. , Okuno, M. , Kurosawa, M. , & Nukina, N. (2011). Serine 403 phosphorylation of p62/SQSTM1 regulates selective autophagic clearance of ubiquitinated proteins. Molecular Cell, 44(2), 279‐289. 10.1016/j.molcel.2011.07.039 22017874

[acel13257-bib-0026] Mizushima, N. , Levine, B. , Cuervo, A. M. , & Klionsky, D. J. (2008). Autophagy fights disease through cellular self‐digestion. Nature, 451(7182), 1069‐1075. 10.1038/nature06639 18305538 PMC2670399

[acel13257-bib-0027] Moldogazieva, N. T. , & Mokhosoev, I. M. (2019). Oxidative Stress and Advanced Lipoxidation and Glycation End Products (ALEs and AGEs) in Aging and Age‐Related Diseases. Oxidative Medicine and Cellular Longevity, 2019, 1–14. 10.1155/2019/3085756 PMC6710759 31485289

[acel13257-bib-0028] Morcos, M. , Du, X. , Pfisterer, F. , Hutter, H. , Sayed, A. A. R. , Thornalley, P. , Ahmed, N. , Baynes, J. , Thorpe, S. , Kukudov, G. , Schlotterer, A. , Bozorgmehr, F. , El Baki, R. A. , Stern, D. , Moehrlen, F. , Ibrahim, Y. , Oikonomou, D. , Hamann, A. , Becker, C. , … Nawroth, P. P. (2008). Glyoxalase‐1 prevents mitochondrial protein modification and enhances lifespan in Caenorhabditis elegans. Aging Cell, 7(2), 260‐269. 10.1111/j.1474-9726.2008.00371.x 18221415

[acel13257-bib-0029] Nenna, A. , Nappi, F. , Avtaar Singh, S. S. , Sutherland, F. W. , Di Domenico, F. , Chello, M. , & Spadaccio, C. (2015). Pharmacologic approaches against advanced glycation end products (AGEs) in diabetic cardiovascular disease. Research in Cardiovascular Medicine, 4(2), e26949 10.5812/cardiovascmed.4(2)2015.26949 26393232 PMC4571620

[acel13257-bib-0030] Ozcelik, S. , Fraser, G. , Castets, P. , Schaeffer, V. , Skachokova, Z. , Breu, K. , Clavaguera, F. , Sinnreich, M. , Kappos, L. , Goedert, M. , Tolnay, M. , & Winkler, D. T. (2013). Rapamycin attenuates the progression of tau pathology in P301S tau transgenic mice. PLoS One, 8(5), e62459 10.1371/journal.pone.0062459 23667480 PMC3646815

[acel13257-bib-0031] Pankiv, S. , Clausen, T. H. , Lamark, T. , Brech, A. , Bruun, J.‐A. , Outzen, H. , Øvervatn, A. , Bjørkøy, G. , & Johansen, T. (2007). p62/SQSTM1 binds directly to Atg8/LC3 to facilitate degradation of ubiquitinated protein aggregates by autophagy. Journal of Biological Chemistry, 282(33), 24131‐24145. 10.1074/jbc.M702824200 17580304

[acel13257-bib-0032] Rabbani, N. , & Thornalley, P. J. (2015). Dicarbonyl stress in cell and tissue dysfunction contributing to ageing and disease. Biochemical and Biophysical Research Communications, 458(2), 221‐226. 10.1016/j.bbrc.2015.01.140 25666945

[acel13257-bib-0033] Rowan, S. , Bejarano, E. , & Taylor, A. (2018). Mechanistic targeting of advanced glycation end‐products in age‐related diseases. Biochimica Et Biophysica Acta (BBA) ‐ Molecular Basis of Disease, 1864(12), 3631‐3643. 10.1016/j.bbadis.2018.08.036 30279139 PMC6822271

[acel13257-bib-0034] Rowan, S. , Jiang, S. , Korem, T. , Szymanski, J. , Chang, M.‐L. , Szelog, J. , Cassalman, C. , Dasuri, K. , McGuire, C. , Nagai, R. , Du, X.‐L. , Brownlee, M. , Rabbani, N. , Thornalley, P. J. , Baleja, J. D. , Deik, A. A. , Pierce, K. A. , Scott, J. M. , Clish, C. B. , … Taylor, A. (2017). Involvement of a gut‐retina axis in protection against dietary glycemia‐induced age‐related macular degeneration. Proceedings of the National Academy of Sciences U S A, 114(22), E4472‐e4481. 10.1073/pnas.1702302114 PMC5465926 28507131

[acel13257-bib-0035] Rubinsztein, D. C. , Marino, G. , & Kroemer, G. (2011). Autophagy and aging. Cell, 146(5), 682‐695. 10.1016/j.cell.2011.07.030 21884931

[acel13257-bib-0036] Schlotterer, A. , Kukudov, G. , Bozorgmehr, F. , Hutter, H. , Du, X. , Oikonomou, D. , Ibrahim, Y. , Pfisterer, F. , Rabbani, N. , Thornalley, P. , Sayed, A. , Fleming, T. , Humpert, P. , Schwenger, V. , Zeier, M. , Hamann, A. , Stern, D. , Brownlee, M. , Bierhaus, A. , … Morcos, M. (2009). C. elegans as model for the study of high glucose‐ mediated life span reduction. Diabetes, 58(11), 2450‐2456. 10.2337/db09-0567 19675139 PMC2768179

[acel13257-bib-0037] Shang, F. , & Taylor, A. (1995). Oxidative stress and recovery from oxidative stress are associated with altered ubiquitin conjugating and proteolytic activities in bovine lens epithelial cells. The Biochemical Journal, 307(Pt 1), 297‐303. 10.1042/bj3070297 7717989 PMC1136776

[acel13257-bib-0038] Simonsen, A. , Cumming, R. C. , Brech, A. , Isakson, P. , Schubert, D. R. , & Finley, K. D. (2008). Promoting basal levels of autophagy in the nervous system enhances longevity and oxidant resistance in adult Drosophila. Autophagy, 4(2), 176‐184. 10.4161/auto.5269 18059160

[acel13257-bib-0039] Spilman, P. , Podlutskaya, N. , Hart, M. J. , Debnath, J. , Gorostiza, O. , Bredesen, D. , & Galvan, V. (2010). Inhibition of mTOR by rapamycin abolishes cognitive deficits and reduces amyloid‐beta levels in a mouse model of Alzheimer's disease. PLoS One, 5(4), e9979 10.1371/journal.pone.0009979 20376313 PMC2848616

[acel13257-bib-0040] Takahashi, A. , Takabatake, Y. , Kimura, T. , Maejima, I. , Namba, T. , Yamamoto, T. , Matsuda, J. , Minami, S. , Kaimori, J.‐Y. , Matsui, I. , Matsusaka, T. , Niimura, F. , Yoshimori, T. , & Isaka, Y. (2017). Autophagy inhibits the accumulation of advanced glycation end products by promoting lysosomal biogenesis and function in the kidney proximal tubules. Diabetes, 66(5), 1359‐1372. 10.2337/db16-0397 28246295

[acel13257-bib-0041] Taylor, A. (2012). Mechanistically linking age‐related diseases and dietary carbohydrate via autophagy and the ubiquitin proteolytic systems. Autophagy, 8(9), 1404‐1406. 10.4161/auto.21150 22906982 PMC3442892

[acel13257-bib-0042] Uchiki, T. , Weikel, K. A. , Jiao, W. , Shang, F. U. , Caceres, A. , Pawlak, D. , Handa, J. T. , Brownlee, M. , Nagaraj, R. , & Taylor, A. (2012). Glycation‐altered proteolysis as a pathobiologic mechanism that links dietary glycemic index, aging, and age‐related disease (in nondiabetics). Aging Cell, 11(1), 1‐13. 10.1111/j.1474-9726.2011.00752.x 21967227 PMC3257376

[acel13257-bib-0043] Vicente Miranda, H. , El‐Agnaf, O. M. , & Outeiro, T. F. (2016). Glycation in Parkinson's disease and Alzheimer's disease. Movement Disorders, 31(6), 782‐790. 10.1002/mds.26566 26946341

[acel13257-bib-0044] Wang, X. J. , Yu, J. , Wong, S. H. , Cheng, A. S. L. , Chan, F. K. L. , Ng, S. S. M. , Cho, C. H. , Sung, J. J. Y. , & Wu, W. K. K. (2013). A novel crosstalk between two major protein degradation systems: regulation of proteasomal activity by autophagy. Autophagy, 9(10), 1500‐1508. 10.4161/auto.25573 23934082

[acel13257-bib-0045] Weikel, K. A. , FitzGerald, P. , Shang, F. U. , Caceres, M. A. , Bian, Q. , Handa, J. T. , Stitt, A. W. , & Taylor, A. (2012). Natural history of age‐related retinal lesions that precede AMD in mice fed high or low glycemic index diets. Investigative Ophthalmology & Visual Science, 53(2), 622‐632. 10.1167/iovs.11-8545 22205601 PMC3317410

[acel13257-bib-0046] Zhang, Y. , Cross, S. D. , Stanton, J. B. , Marmorstein, A. D. , Le, Y. Z. , & Marmorstein, L. Y. (2017). Early AMD‐like defects in the RPE and retinal degeneration in aged mice with RPE‐specific deletion of Atg5 or Atg7. Molecular Vision, 23, 228‐241. 28465655 PMC5398883

