# Supplementary figures and images for: Autophagic receptor p62 protects against glycation‐derived toxicity and enhances viability
Source: Aging Cell. 2020 Nov 4;19(11):e13257. doi: 10.1111/acel.13257 (PMC7681057; doi:10.1111/acel.13257)

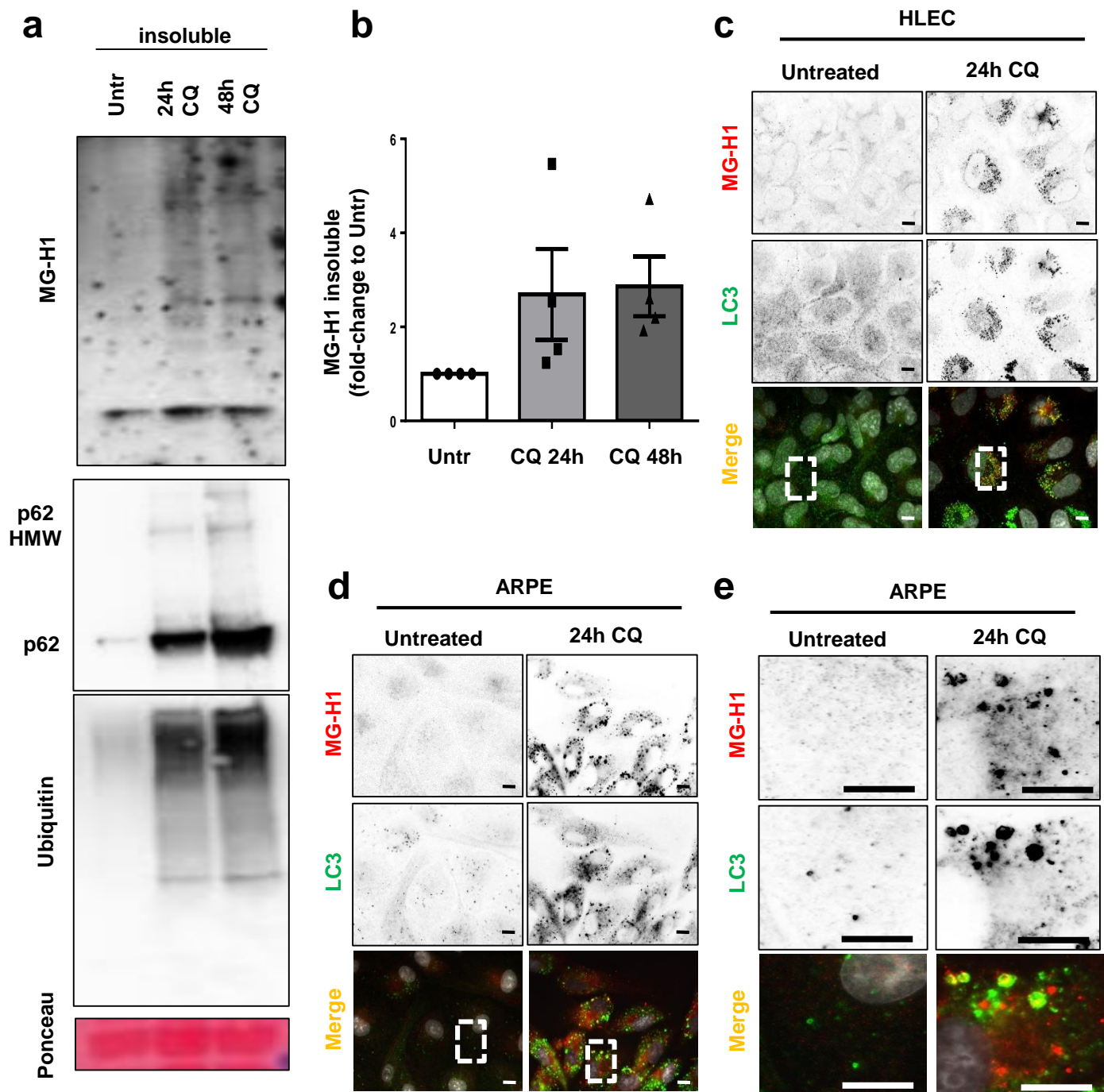

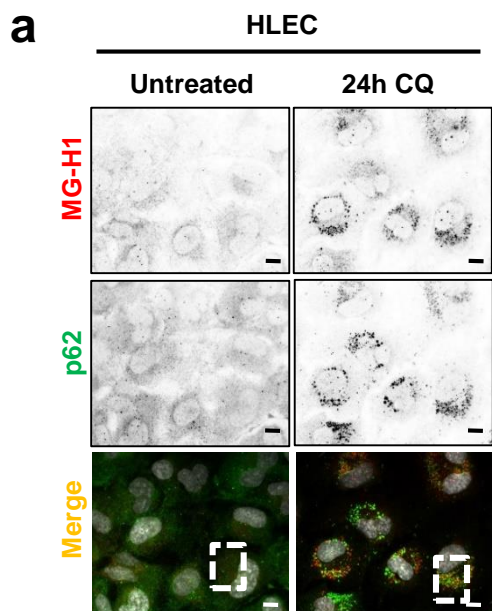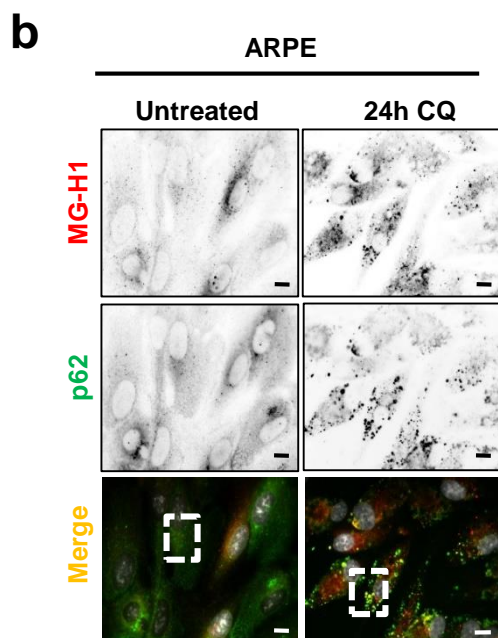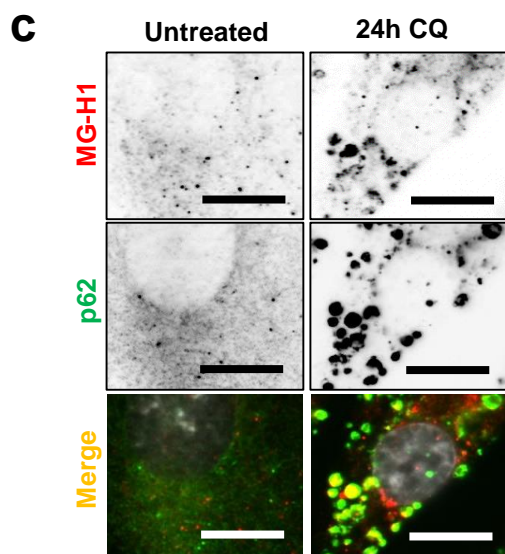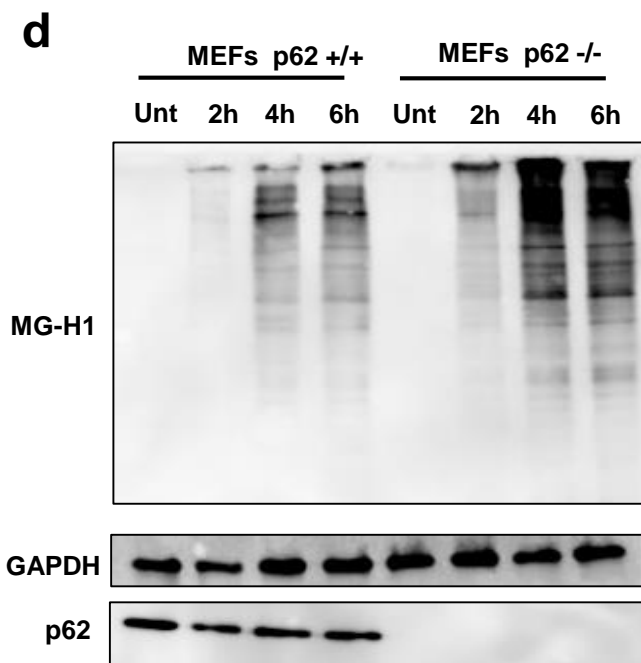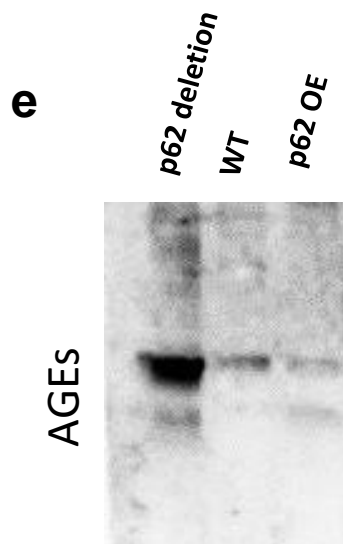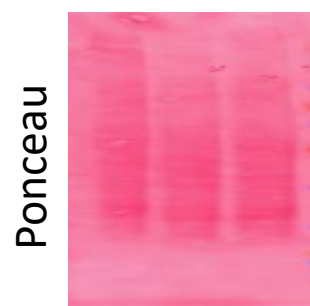

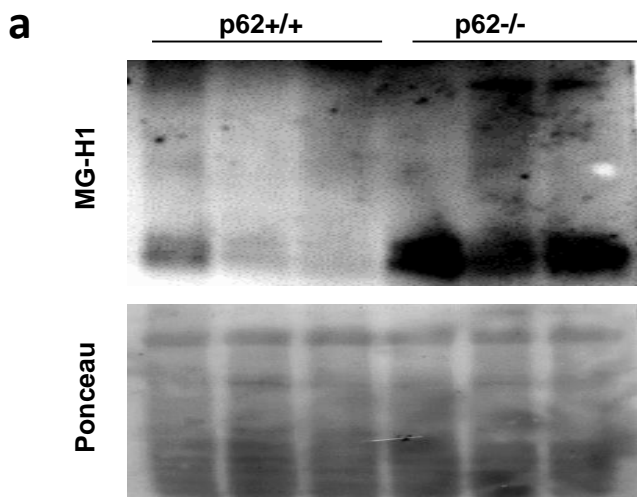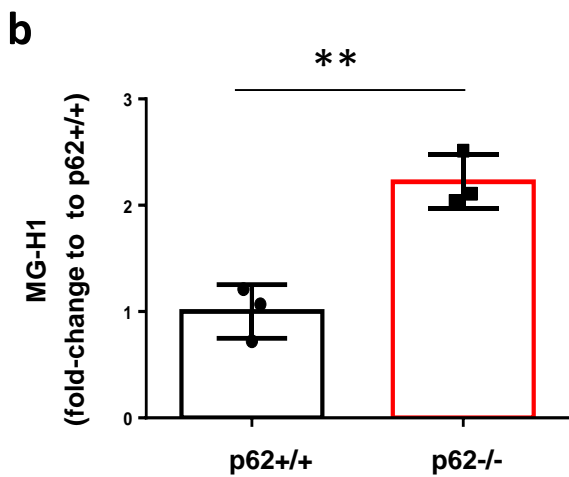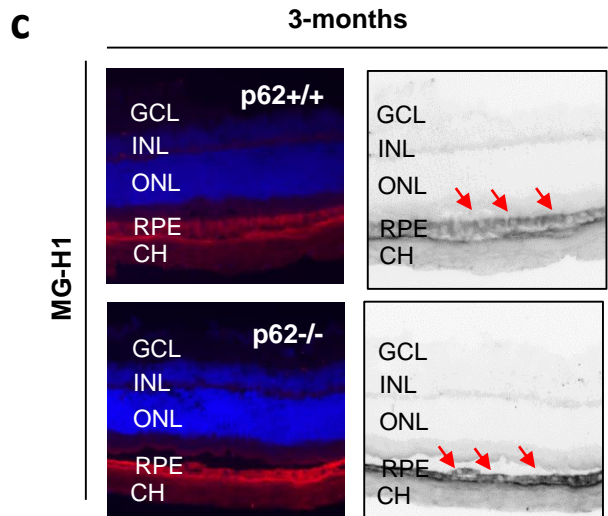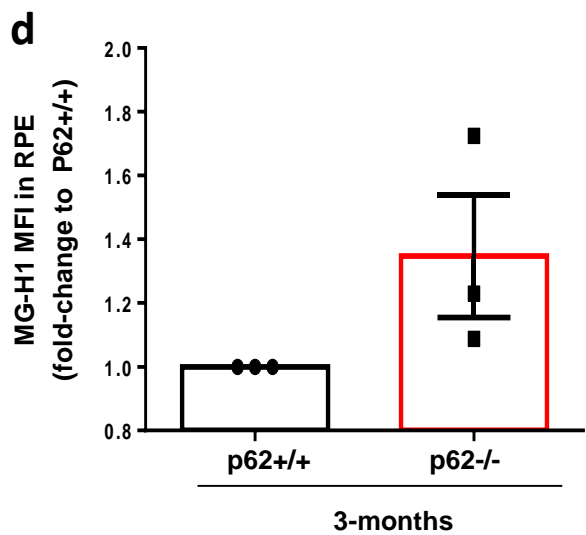

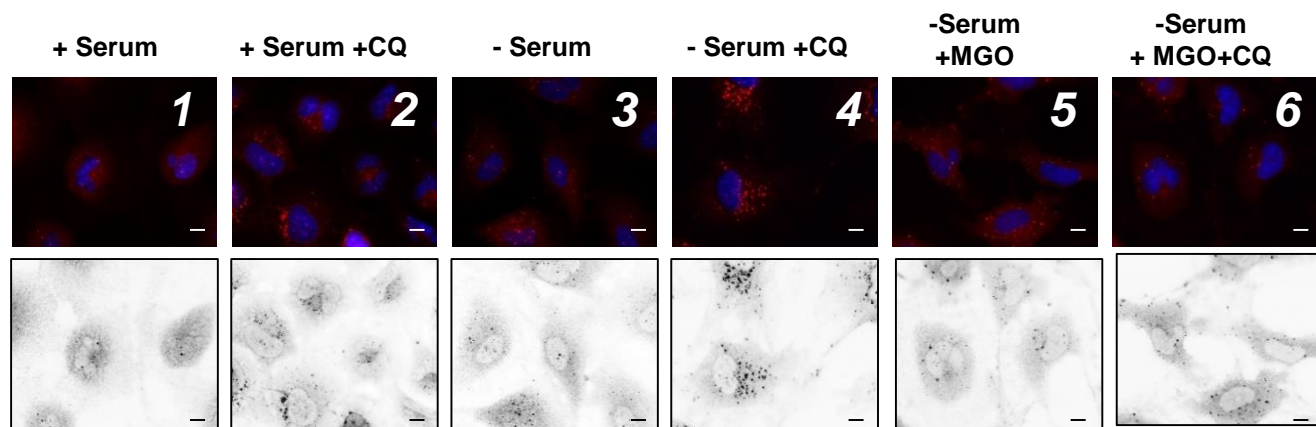

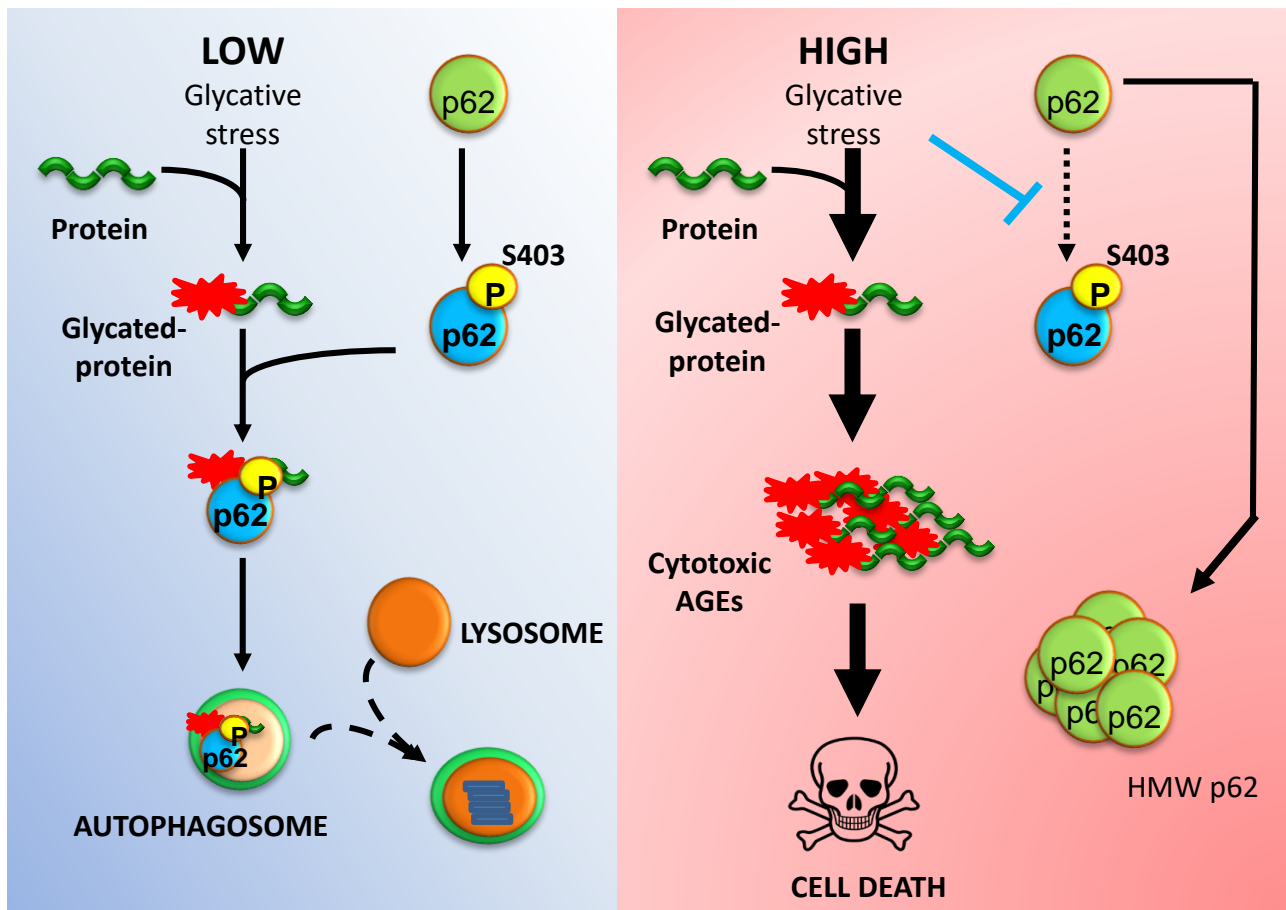

Supplement: Supplementary file 1 [file ACEL-19-e13257-s001.pdf]
