# Supplementary material for: Autophagic receptor p62 protects against glycation‐derived toxicity and enhances viability
Source: Aging Cell. 2020 Nov 4;19(11):e13257. doi: 10.1111/acel.13257 (PMC7681057; doi:10.1111/acel.13257)
Supplement: Supplementary file 2 [file ACEL-19-e13257-s002.docx]

Supporting Informationfor

**Autophagic receptor p62 protects against glycation-derived toxicity and enhances viability**

Gemma Aragonès^1^, Kalavathi Dasuri^1^, Opeoluwa Olukorede^1^, Sarah G Francisco^1^, Carol Renneburg^1^, Caroline Kumsta^2^, Malene Hansen^2^, Shun Kageyama^3^, Masaaki Komatsu^3^,Sheldon Rowan^1^, Jonathan Volkin^1^, Michael Workman^1^, Wendy Yang^1^,Paula Daza^4^, Diego Ruano^5,6^, Helena Dominguez-Martín^5,6^ José Antonio Rodríguez-Navarro^7^,Xue-Liang Du^8^, Michael A. Brownlee^8^, Eloy Bejarano^1*^, Allen Taylor^1*^

1 Laboratory for Nutrition and Vision Research, USDA Human Nutrition Research Center on Aging, Tufts University, Boston, USA.

2 Sanford Burnham Prebys Medical Discovery Institute, La Jolla, CA 92037, USA.

3Department of Physiology, Juntendo University School of Medicine, Japan.

4 Departamento Biología Celular. Facultad de Biología. Universidad de Sevilla, Spain.

5 Departamento de Bioquímica y Biología Molecular. Facultad de Farmacia. Universidad de Sevilla, Spain.

6 Instituto de Biomedicina de Sevilla (IBiS), Hospital Universitario Virgen del Rocío/Consejo Superior de Investigaciones Cientíﬁcas/Universidad de Sevilla, Sevilla, Spain.

7 Servicio de Neurobiología, Departamento de Investigación, Hospital Ramón y Cajal, Instituto Ramón y Cajal de Investigaciones Sanitarias, Carretera de Colmenar, km 9,100, Madrid, Spain.

8. Albert Einstein College of Medicine, Bronx, NY, USA.

* Corresponding authors

**Eloy Bejarano**, Laboratory for Nutrition and Vision Research, USDA Human Nutrition Research Center on Aging, Tufts University, Phone: 617 556 3158; Fax 617 556 3132; E-mail: [eloy.bejarano@tufts.edu](mailto:eloy.bejarano@tufts.edu)

**Allen Taylor**, Laboratory for Nutrition and Vision Research, USDA Human Nutrition Research Center on Aging, Tufts University, Phone: 617 556 3156; Fax 617 556 3132; E-mail: [allen.taylor@tufts.edu](mailto:allen.taylor@tufts.edu)

**Keywords**

Glycative stress, Proteotoxicity, Autophagy, Aging, p62

**This PDF file includes:**

Figures S1 to S5

**Fig. S1. Autophagy plays a critical role in the removal of endogenous AGEs.(a,b)** Lysates from HLECs maintained in the presence or absence of CQ for either 24h or 48h wer e subjected to extraction with 1% Triton X-100. **(a)** Insoluble fractions were immunoblotted for the indicated proteins and **(b)** quantification of insoluble AGEs. Values are mean ± SEM (n = 4) **(c)** HLECs were maintained in the presence or absence of CQ for 24h, fixed in cold methanol and anti-LC3 (green) and anti MG-H1 (red) were used to detect endogenous proteins. Red and green channels are shown in black and white in the upper panels for a better visualization.The areas within the white squares in this 20x are the magnified areas shown in Main Figure 1e.**(d,e)** Same co-localization analysis was carried out in ARPE-19. **(d)** 20x representative pictures and **(e)** magnified areas to illustrate the accumulation of MG-H1 in ARPE-19 in autophagosomes. Scale bar: 10 μm.

**Fig. S2. p62 contribution in AGEs clearance**. **(a)** HLECs were maintained in the presence or absence of CQ for 24h, fixed in cold methanol and anti-LC3 (green) and anti MG-H1 (red) were used to detect endogenous proteins. Red and green channels are shown in black and white in the upper panels for a better visualization.The area within the white squares in this 20x representative pictures are the magnified areas shown in Main Figure 1f.**(b)** Same co-localization analysis was carried out in ARPE-19. **(c)** magnified areas to illustrate the accumulation of MG-H1 in autophagosomes in ARPE-19 are shown.Scale bar: 10 μm. **(d)** Representative immunoblot for MG-H1 in whole cellular extract from WT MEFs (p62+/+) and MEFs lacking p62 (p62-/-) incubated with 1mM MGO for the indicated times. **(e)** Representative immunoblot for AGEs in *C*.*elegans*. Samples are from p62 deletion mutants, WT, and animals overexpressing p62.

**Fig. S3. Lack of p62 induces accumulation of AGEs *in vivo*.(a,b)** Immunoblot for MG-H1 in liver tissues from WT and whole body p62-/- mice. **(a)** Representative immunoblot and **(b)** quantification of total levels of MG-H1 relative to values in WT. Values are mean ± SEM (n = 3) **p <0.01**(c,d)** Immunohistochemistry for MG-H1 in retinal tissues from 3 month-old p62+/+ and whole body p62-/- mice. **(c)** 20x representative pictures and **(d)** quantification of MFI relative to values in p62+/+ are shown. Arrows point out the retinal pigment epithelial layer (RPE). Values are mean ± SEM (n = 4). Abbreviations: CH, choroid; RPE, retinal pigment epithelium; INL, inner nuclear layer; IPL, inner plexiform layer; ONL, outer nuclear layer; GCL, ganglion cell layer.

Fig. S4. Lysosomal targeting of p62 is compromised under glycative stress. HLECs cells were maintained in complete medium (+S) or serum-free medium (-S) in presence/absence of 2mM MGO, 30μM chloroquine (CQ) or combination of both for 2 hours, fixed and immunostained against endogenous p62. Black and white pictures in the bottom panels are shown for a better visualization. Quantitative parameters of this analysis are shown in Main Figure 4.

**Figure S5. Hypothetical model of the protective role of p62 against glycation-derived damage.** Under low glycative stress conditions (*left*), AGEs are formed by non-enzymatic reactions between sugars and proteins. Larger or insoluble AGEs that are poorly degraded by the proteasome are recognized by the autophagic receptor p62. p62 phosphorylation at S403 regulates p62 targeting to the autophagosome and, consequently, the autophagic clearance of AGEs. Under high glycative stress (*right*), phosphorylation of p62 at S403 diminishes, resulting in the accumulation of HMW-p62, and glycated proteins are accumulated leading to cell death
